# Roles of Type 1A Topoisomerases in Genome Maintenance in *Escherichia coli*

**DOI:** 10.1371/journal.pgen.1004543

**Published:** 2014-08-07

**Authors:** Valentine Usongo, Marc Drolet

**Affiliations:** Département de microbiologie, infectiologie et immunologie, Université de Montréal, Succ. Centre-ville, Montréal, Québec, Canada; Institute of Molecular and Cell Biology (IMCB), A*STAR, Singapore

## Abstract

In eukaryotes, type 1A topoisomerases (topos) act with RecQ-like helicases to maintain the stability of the genome. Despite having been the first type 1A enzymes to be discovered, much less is known about the involvement of the *E. coli* topo I (*topA*) and III (*topB*) enzymes in genome maintenance. These enzymes are thought to have distinct cellular functions: topo I regulates supercoiling and R-loop formation, and topo III is involved in chromosome segregation. To better characterize their roles in genome maintenance, we have used genetic approaches including suppressor screens, combined with microscopy for the examination of cell morphology and nucleoid shape. We show that *topA* mutants can suffer from growth-inhibitory and supercoiling-dependent chromosome segregation defects. These problems are corrected by deleting *recA* or *recQ* but not by deleting *recJ* or *recO*, indicating that the RecF pathway is not involved. Rather, our data suggest that RecQ acts with a type 1A topo on RecA-generated recombination intermediates because: 1-topo III overproduction corrects the defects and 2-*recQ* deletion and topo IIII overproduction are epistatic to *recA* deletion. The segregation defects are also linked to over-replication, as they are significantly alleviated by an *oriC*::*aph* suppressor mutation which is *oriC*-competent in *topA* null but not in isogenic *topA^+^* cells. When both topo I and topo III are missing, excess supercoiling triggers growth inhibition that correlates with the formation of extremely long filaments fully packed with unsegregated and diffuse DNA. These phenotypes are likely related to replication from R-loops as they are corrected by overproducing RNase HI or by genetic suppressors of double *topA rnhA* mutants affecting constitutive stable DNA replication, *dnaT*::*aph* and *rne*::*aph*, which initiates from R-loops. Thus, bacterial type 1A topos maintain the stability of the genome (i) by preventing over-replication originating from *oriC* (topo I alone) and R-loops and (ii) by acting with RecQ.

## Author Summary

DNA topoisomerases are ubiquitous enzymes that solve the topological problems associated with replication, transcription and recombination. Eukaryotic enzymes of the type 1A family work with RecQ-like helicases such as BLM and Sgs1 and are involved in genome maintenance. Interestingly, *E. coli* topo I, a type 1A enzyme and the first topoisomerase to be discovered, appears to have distinct cellular functions that are related to supercoiling regulation and to the inhibition of R-loop formation. Here we present data strongly suggesting that these cellular functions are required to inhibit inappropriate replication originating from either *oriC*, the normal origin of replication, or R-loops that can otherwise lead to severe chromosome segregation defects. Avoiding such inappropriate replication appears to be a key cellular function for genome maintenance, since the other *E. coli* type 1A topo, topo III, is also involved. Furthermore, our data suggest that bacterial type 1A topos, like their eukaryotic counterparts, can act with RecQ in genome maintenance. Altogether, our data provide new insight into the role of type 1A topos in genome maintenance and reveal an interplay between these enzymes and R-loops, structures that can also significantly affect the stability of the genome as recently shown in numerous studies.

## Introduction

Type 1A topoisomerases (topos) are essential and ubiquitous enzymes found in bacteria, archaea and eukarya [1], [2]. They all require single-stranded DNA (ssDNA) regions for activity. Such substrates can already be present, for example, in negatively supercoiled DNA, at the replication fork and in R-loops, or can be generated by the action of proteins, such as helicases or RNA polymerases. *E. coli* topo I, the first topo to be discovered [3], is the prototype enzyme of this family. This enzyme binds to ssDNA close to double-stranded DNA (dsDNA) regions [4] and is therefore well suited to relax the excess negative supercoiling generated behind RNA polymerase molecules during transcription [5], or introduced by DNA gyrase, the enzyme that negatively supercoils DNA in bacteria [6].

The best evidence for a major role of topo I in the regulation of supercoiling came from the observation that *topA* null mutants accumulate compensatory mutations in *gyrA* or *gyrB* genes allowing them to grow [7]. These mutations decrease the supercoiling activity of gyrase which leads to a reduction in the global chromosome supercoiling level below that of wild-type cells [8]. The role of topo I in transcription is supported by the finding that it physically interacts with RNA polymerase [9]. One major consequence of excess negative supercoiling is R-loop formation [10]. This is supported by the observation that the growth defect of *topA* null mutant can be partially compensated by RNase HI overproduction [11]. Evidence for extensive R-loop formation in the absence of topo I has been provided both *in vitro* and *in vivo* [12]–[15]. It is believed that topo I prevents R-loop formation mainly by relaxing transcription-induced negative supercoiling [15].

After a temperature downshift to reactivate gyrase in a *topA* null mutant carrying a *gyrB*(Ts) allele, RNase HI overproduction was shown to prevent a transient growth arrest that correlated with the accumulation of excess negative supercoiling (hypernegative supercoiling) and extensive RNA degradation [16]. RNase HI overproduction was found both to reduce the accumulation of excess negative supercoils and to promote their rapid removal by topo IV [16], [17], the other enzyme that can relax negative supercoiling in *E. coli* [18]. Moreover, evidence for R-loops impeding transcription of ribosomal RNA genes (*rrn* operons) in *topA* null mutants has been reported [19]. Interestingly, R-loop-dependent gene expression inhibition related to RNA polymerase arrest and RNA degradation has also been reported for yeast cells lacking topo I, a type 1B topo [20]. Thus, R-loop-mediated impairment of gene expression appears to be a major mechanism by which excess negative supercoiling inhibits growth.

*E. coli* topo I is a relatively abundant protein being in the top 25% of the most abundant proteins in *E. coli* (134 ppm) [21]. The *topA* gene is under the control of promoters recognized by different sigma factors, σ^32^, σ^S^ and σ^70^ and its expression is important for *E. coli*'s response to various stresses including heat and oxidative shock [22]. RNase HI overproduction was shown to partially restore the expression of σ^32^-regulated genes required for the heat shock response [23].

Although studies of topo I mostly focused on its role in supercoiling regulation and its effect on gene expression, evidence for the involvement of this enzyme in other DNA transactions such as chromosome segregation and replication initiation has been provided [17], [24]–[27]. Interestingly, one of the first functions to be proposed for topo I was a role as a specificity factor to inhibit replication initiation outside *oriC*, such as initiation from R-loops, that could occur in an *in vitro* reconstituted system for *oriC*-dependent replication [28]. However, there is no experimental evidence for such a role of topo I *in vivo*.

*E. coli* topo III, the second type 1A topo to be discovered, has a much higher preference for ssDNA than *E. coli* topo I [29]. As a consequence, topo III is very inefficient in relaxing DNA with a physiological supercoiling density and, in fact, this enzyme plays no role in supercoiling regulation [18], [30]. However, topo III was shown to be a very potent decatenase during replication *in vitro* provided that a ssDNA region was present on the DNA substrate for the binding of the enzyme [29]. The presence of a unique amino-acid sequence in the topo III protein named the “decatenation loop” (absent in eukaryotic type 1A enzymes), was found to be essential for the decatenation of replication intermediates [31].

Unlike topo I, topo III is a protein of low abundance (9.4 ppm) [21]. Moreover, as opposed to *topA* null mutants, cells lacking topo III activity display no growth defects [32]. Recently, it has been shown that topo III plays a role in chromosome segregation *in vivo* that is likely related to replication, as this function was shown to be mostly required when the activity of topo IV [33], the main cellular decatenase, or gyrase [34] was severely impaired. Topo III physically interacts with SSB protein and this interaction presumably allows topo III to act at the replication fork in the cell [35].

Similar to eukaryotic type 1A topos (see below), topo III activity was shown to be stimulated by RecQ helicase *in vitro* [35]–[37], but these two proteins do not physically interact. Evidence for RecQ acting with topo III in *E. coli* cells has been reported [30]. However, because some important properties of the strains used in this work could not be observed in an independent study, the conclusion that RecQ acts with topo III has been questioned [33].

*Saccharomyces cerevisiae* type 1A topo was the first enzyme of this family to be discovered in eukaryotic cells [38]. Being the third topo identified in this organism, it was named Top3. The existence of this topo was revealed following the isolation of a mutation, in *top3*, that stimulated recombination between repeated sequences [38]. Interestingly, phenotypes of *top3* mutants including slow growth and sporulation deficiency were suppressed to different extents by inactivating *SGS1*, encoding the RecQ homolog of *S. cerevisiae*, or by overproducing *E. coli* topo I [38]–[40]. Moreover, deleting *RAD51*, encoding the RecA homolog of *S. cerevisiae*, was shown to rescue the slow growth phenotype of *top3* mutants [41]. Altogether, these data suggested that Sgs1 processed recombination intermediates to generate structures that could only be resolved by a type 1A topo, such as Top3 or *E. coli* topo I.

Physical interactions between type 1A topos (named topo III in higher eukaryotes) and their RecQ-like partner from eukaryotic organisms (e.g BLM in humans and in *Drosophila*) have been demonstrated [1], [39], [42], [43]. It is now well established that these complexes can efficiently resolve homologous recombination intermediates (Double Holliday Junctions; DHJs) without genetic exchange [1], [44]–[46]. Reactions of BLM with topo III are often stimulated by the presence of RPA, the SSB homolog of eukaryotes that presumably stabilizes the BLM-generated ssDNA region, the substrate for topo III [44], [46]. Eukaryotic topo III enzymes have a higher requirement for ssDNA than *E. coli* topo I and, in fact, they are generally considered to be more closely related to *E. coli* topo III than topo I [1].

In *E. coli*, an interplay between topo I and III has been reported in two instances. In the first one, the *topB* gene was isolated as a multicopy suppressor of a *topA* null mutant [47]. Despite the significant correction of the growth defect of the *topA* null mutant by overproducing topo III, relaxation of the excess negative supercoiling introduced by gyrase was barely detected. This is consistent with our observation that topo III overproduction, unlike RNase HI overproduction, is unable to prevent the supercoiling-dependent transient growth arrest of a *topA gyrB*(Ts) strain, following a temperature downshift ([16]; Baaklini and Drolet, unpublished). These results might have suggested that topo III overproduction complemented a yet unknown function of topo I that was not directly related to excess supercoiling. Indeed, here we present genetic evidence for an important role of topo I acting with RecQ to resolve RecA-dependent recombination intermediates that otherwise inhibit chromosome segregation. Moreover, our data suggest that the requirement for this activity is related to over-replication mostly from *oriC* that takes place in the absence of *topA*, presumably due to excess negative supercoiling.

In the second instance, deleting *topB* from a *topA* null mutant carrying a *gyrA* or *gyrB* compensatory mutation, led to the formation of very long filaments with unsegregated nucleoids having abnormal structures and, eventually, to growth arrest [48]. Here, our data suggest that these phenotypes are exacerbated by excess negative supercoiling and are mostly related to over-replication from R-loops.

Overall, our data demonstrate that bacterial type 1A topos maintain the stability of the genome by preventing unregulated replication and at least one of its consequences, namely the inhibition of chromosome segregation.

## Results

### Supercoiling-dependent growth and chromosome segregation defects in cells lacking topo I activity

**Figure 1:**
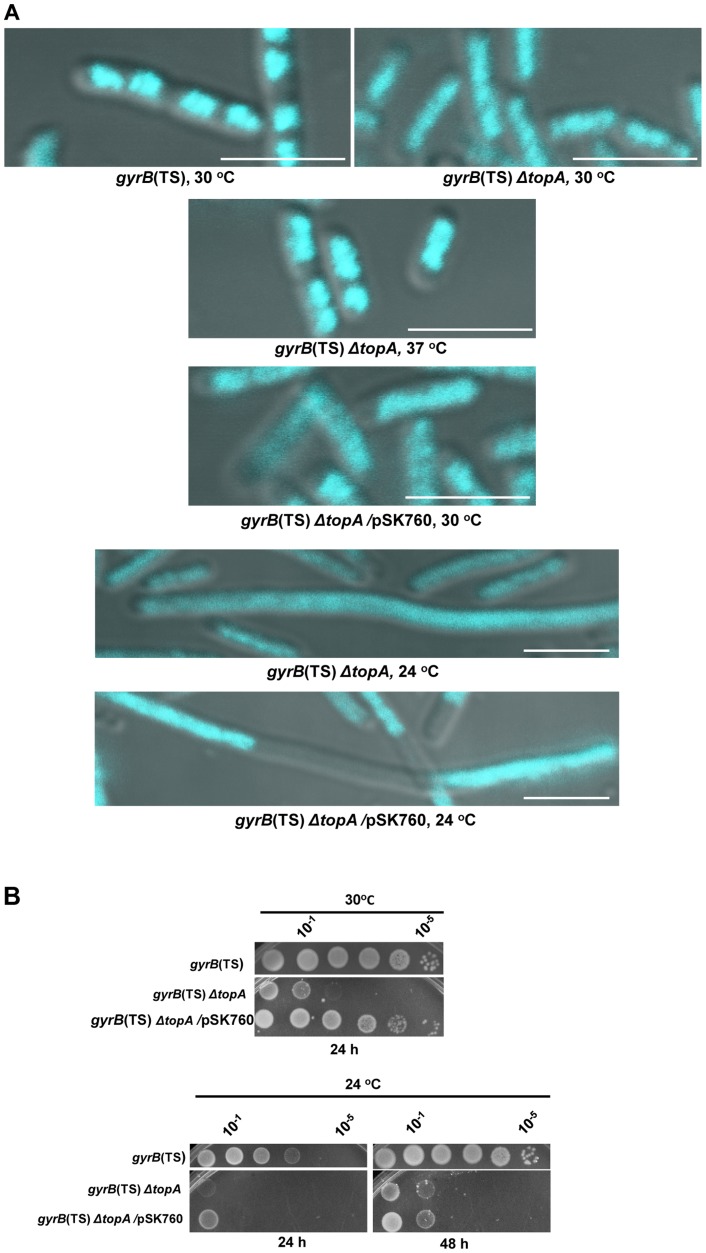
Growth and chromosome segregation defects in the *gyrB*(Ts) *ΔtopA* strain. (a) Representative superimposed images of DIC and fluorescence pictures of DAPI-stained cells grown at the indicated temperatures, as described in Materials and Methods. Size bars are 5 µm. Additional images are shown in Figure S1 and S2. (b) Spot tests were performed at the indicated temperatures. The LB plates were incubated for the indicated times. The strains used are RFM475 (*gyrB*(Ts) *ΔtopA*), RFM445 (*gyrB*(Ts)) and VU287 (RFM475/pSK760). pSK760 carries the *rnhA* gene for RNase HI overproduction.

To look for chromosome segregation defects in a *topA* null mutant, cells of a *ΔtopA gyrB*(Ts) strain were stained with DAPI and prepared for microscopy such that both cell morphology and DNA content could be examined. By growing the cells at 30°C, the permissive temperature for gyrase, we could test the true effect of losing *topA* on nucleoid shape. As can be seen in Figure 1A, whereas nucleoids of *gyrB*(Ts) cells were well separated and compact, those of isogenic *gyrB*(Ts) *ΔtopA* cells were less compact and clearly not separated, thus showing chromosome segregation defects.

To verify if these problems were related to excess negative supercoiling, *topA* null cells were grown at 37°C so that gyrase activity was reduced. At this temperature the chromosome supercoiling level decreases below that of wild-type cells and, as a result, *topA* null cells can grow robustly [11], [49]. At 37°C, chromosome segregation in the *gyrB*(Ts) *ΔtopA* strain was significantly improved, as many cells had well separated and more compact nucleoids as compared to cells grown at 30°C (Figure 1A, *gyrB*(Ts) *ΔtopA*, 37°C vs 30°C). We tested the effect of RNase HI overproduction on chromosome segregation in the *gyrB*(Ts) *ΔtopA* strain grown at 30°C. It did not correct the chromosome segregation defect (*gyrB*(Ts) *ΔtopA*/pSK760). Thus, we conclude that *topA* null cells suffer from supercoiling-dependent chromosome segregation defects that are unrelated to R-loops.

RNase HI overproduction did not correct the chromosome segregation problem whereas it clearly stimulated the growth of *gyrB*(Ts) *ΔtopA* cells at 30°C (Figure 1B, *gyrB*(Ts) *ΔtopA* vs *gyrB*(Ts) *ΔtopA*/pSK760). Therefore, at this temperature the defect was not strong enough to offset the positive effect of overproducing RNase HI. We have previously shown that RNase HI overproduction could not complement the growth defect of *topA* null mutants at lower temperatures. In fact, it had a negative effect [47], [50]. The cold sensitivity of *topA* null mutants was found to be, at least in part, related to the inability of topo IV to efficiently relax negative supercoiling at low temperatures [16], [17]. As a result, hypernegative supercoiling accumulated.

We found that the chromosome segregation defect of our *gyrB*(Ts) *ΔtopA* strain was exacerbated at 24°C since the cells were generally longer and the DNA more diffuse as compared to cells grown at 30°C (Figure 1A, *gyrB*(Ts) *ΔtopA*, 30°C vs 24°C). Overproducing RNase HI further stimulated cell filamentation and produced cells with large DNA-free regions (Figure 1A, *gyrB*(Ts) *ΔtopA*/pSK760, 24°C). Growth of *gyrB*(Ts) *ΔtopA* cells on solid LB medium at 24°C was very poor whether RNase HI was overproduced or not (Figure 1B, 24°C). Thus, the cold sensitivity of *topA* null cells triggered by excessive hypernegative supercoiling correlates with a strong chromosome segregation defect that seems to be exacerbated by RNase HI overproduction.

### Topo III overproduction and *recA* or *recQ* deletions correct both the growth and chromosome segregation defects in cells lacking topo I activity

Topo III overproduction was previously shown to correct the growth defect of *topA* null mutants at low temperatures [47]. In fact, unlike RNase HI, topo III overproduction was able to correct the growth defect of *gyrB*(Ts) *ΔtopA* cells at 21°C [47]. Since topo III is a potent decatenase and because the growth defect of *gyrB*(Ts) *ΔtopA* cells at 24°C correlates with a strong chromosome segregation problem (Figure 1A), topo III overproduction may have complemented by correcting this segregation defect.

**Figure 2:**
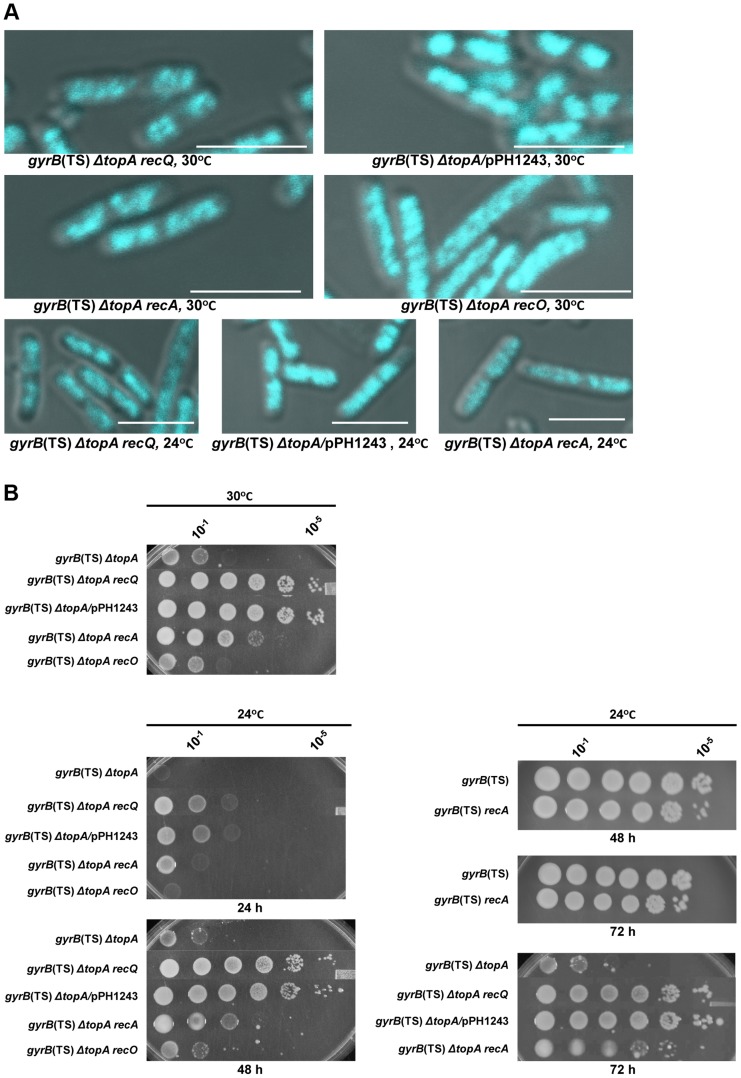
Topo III overproduction, and *recA* and *recQ* deletions complement the growth and chromosome segregation defects in the *gyrB*(Ts) *ΔtopA* strain. (a) Representative superimposed images of DIC and fluorescence pictures of DAPI-stained cells grown at the indicated temperatures as described in Materials and Methods. Size bars are 5 µm. Additional images are shown in Figure S1 and S2. (b) Spot tests were performed at the indicated temperatures. The LB plates were incubated for the indicated times. The strains used are all derivatives of RFM475 (*ΔtopA gyrB(*Ts)) except SB264 which is a derivative of RFM445 (*gyrB*(Ts)). They are: CT150 (RFM475 *ΔrecQ*), VU118 (RFM475/pPH1243), SB265 (RFM475 *ΔrecA*), VU454 (RFM475 *ΔrecO*) and SB264 (RFM445 *ΔrecA*). Cells carrying pPH1243 were grown in the presence of IPTG to overproduce topo III.

This was confirmed by the observation that overproducing topo III almost fully, at 30°C, or partially, at 24°C, corrected the chromosome segregation defect of *gyrB*(Ts) *ΔtopA* cells (*gyrB*(Ts) *ΔtopA*/pPH1243; Figure 2A, at 30°C the nucleoids are well separated and compact; at 24°C some nucleoids separated, shorter cells and the DNA is more compact as compared to cells not overproducing topo III). As expected, topo III overproduction also promoted growth on solid media at these temperatures (Figure 2B). Thus, topo III overproduction corrects the growth defect of *topA* null mutants, at least in part, by facilitating chromosome segregation.

The next series of experiments were performed to test the hypothesis that, as is the case in eukaryotic cells, *E. coli* type 1A topos can act with RecQ to resolve RecA-generated recombination intermediates. We first tested the effect of deleting *recQ* on growth and chromosome segregation in *topA* null cells. We found that deleting *recQ* was as good as overproducing topo III in correcting the growth defect of our *gyrB*(Ts) *ΔtopA* strain at both 30 and 24°C (Figure 2B; *gyrB*(Ts) *ΔtopA ΔrecQ*; western blot experiments showed that topo IV was not overproduced in the *topA* null strain lacking *recQ*; Figure S3). Deleting *recQ* also partially corrected the chromosome segregation defect at these temperatures (Figure 2A). Thus, our results suggest that *recQ* and *topB* act in a pathway that is related to chromosome segregation in the absence of *topA*.

We next tested the effect of deleting *recA* on growth and chromosome segregation in *topA* null cells. The deletion of *recA* partially corrected the growth defect of our *gyrB*(Ts) *ΔtopA* strain at both 30 and 24°C (Figure 2B; *gyrB*(Ts) *ΔtopA ΔrecA*), though the effect was not as good as the one conferred by deleting *recQ* or overproducing topo III (Figure 2B). In fact, the positive effect of deleting *recA* on the growth of *topA* null cells at 24°C was more readily observed after three days of incubation (Figure 2B, 72 h). Deleting *recA* also partially alleviated the chromosome segregation defects of *topA* null cells at both temperatures (Figure 2A). These results demonstrate that the chromosome segregation defects of *topA* null mutants are largely RecA-dependent and therefore support the involvement of homologous recombination.

In *E. coli*, positive effects of deleting *recQ* on growth and chromosome segregation are often attributed to unnecessary RecA-mediated recombination via the RecFOR pathway at arrested replication forks [51], [52]. In this pathway, RecQ helicase acts with RecJ, a 5′-3′ exonuclease, to provide ssDNA regions on which RecF, O and R facilitate RecA nucleoprotein filament assembly. We found that deleting *recJ*, *recO* or *recR* had no effect on growth and chromosome segregation in our *gyrB*(Ts) *ΔtopA* strain (Figure 2, *gyrB*(Ts) *ΔtopA ΔrecO*; data not shown for *recJ* and *recR*). This indicated that the RecFOR pathway was not involved and therefore may suggest that RecQ and type 1A topos act together in a RecA-dependent recombination pathway.

**Figure 3:**
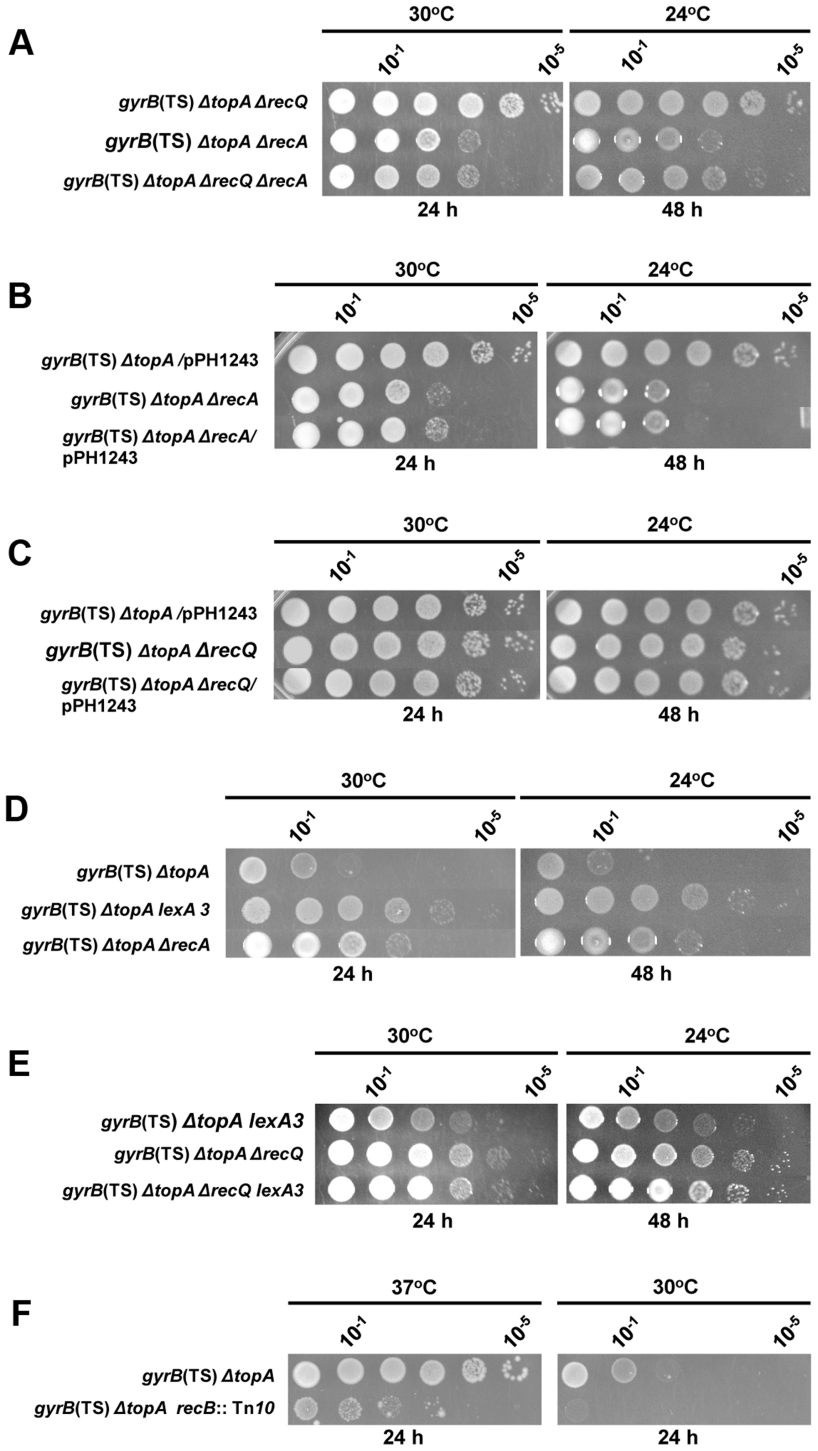
Topo III overproduction and *recQ* deletion are epistatic to *recA* deletion in correcting the growth defect of the *topA gyrB*(Ts) strain. Spot tests were performed at the indicated temperatures. The LB plates were incubated for the indicated times. The strains used are all derivatives of RFM475 (*gyrB*(Ts) *ΔtopA*). They are: a) CT150 (RFM475 *ΔrecQ*), SB265 (RFM475 *ΔrecA*) and VU492 (*ΔrecQ ΔrecA*); b) VU118 (RFM475/pPH1243), SB265 (RFM475 *ΔrecA*) and VU479 (SB265/pPH1243); c) VU118 (RFM475/pPH1243), CT150 (RFM475 *ΔrecQ*) and VU464 (CT150/pPH1243); d) RFM475, SB362 (RFM475 *lexA3*) and SB265 (RFM475 *ΔrecA*); e) SB362 (RFM475 *lexA3*), CT150 (RFM475 *ΔrecQ*) and VU501 (RFM475 *ΔrecQ lexA3*); f) RFM475 and SB262 (RFM475 *recB*::Tn*10*). Cells carrying pPH1243 were grown in the presence of IPTG to overproduce topo III.

If indeed RecQ acts on RecA-generated recombination intermediates to generate substrates for type 1A topos, neither topo III overproduction nor *recQ* deletion should improve the growth of *gyrB*(Ts) *ΔtopA* cells lacking *recA*. Moreover, overproducing topo III should also have no effects on the growth of *gyrB*(Ts) *ΔtopA ΔrecQ* cells. To test these predictions, the appropriate strains were constructed and spot assays were performed. As predicted, combinations of *recQ* and *recA* deletions or of topo III overproduction and *recA* mutation resulted in the same growth phenotype as the *recA* mutation alone (Figure 3A, compare *gyrB*(Ts) *ΔtopA ΔrecQ*, *gyrB*(Ts) *ΔtopA ΔrecA* and *gyrB*(Ts) *ΔtopA ΔrecQ ΔrecA*; Figure 3B, compare *gyrB*(Ts) *ΔtopA*/pPH1243, *gyrB*(Ts) *ΔtopA ΔrecA* and *gyrB*(Ts) *ΔtopA ΔrecA*/pPH1243). Furthermore, topo III overproduction did not improve the growth of *gyrB*(Ts) *ΔtopA ΔrecQ* cells (Figure 3C). These results are consistent with RecQ processing RecA-generated recombination intermediates in such a way that they can only be resolved by a type 1A topo. Since topo III needs to be overproduced, we believe that the much more abundant topo I enzyme is normally involved in the resolution of these intermediates (see Discussion).

Our microarray results indicated that the SOS response was constitutively expressed in our *gyrB*(Ts) *ΔtopA* strain and therefore that RecA was overproduced (not shown). The *lexA3* allele was used to test the effect of the SOS response on the growth of the *gyrB*(Ts) *ΔtopA* strain. This allele makes the SOS response non-inducible and therefore considerably reduces the amount of RecA proteins produced. The *lexA3* allele was found to be slightly better than deleting *recA* to improve the growth of the *gyrB*(Ts) *ΔtopA* strain (Figure 3D, compare *gyrB*(Ts) *ΔtopA*, *gyrB*(Ts) *ΔtopA lexA3* and *gyrB*(Ts) *ΔtopA ΔrecA*; several *lexA3* transductants were tested and were found to behave the same way). This result may suggest that the major effect of the *recA* mutation on the growth of the *gyrB*(Ts) *ΔtopA* strain was not related to the silencing of the SOS response but rather to the inactivation of the recombination function of RecA. If this is true, the *lexA3* allele should behave differently from the *recA* mutation when combined with the *recQ* mutation in the *gyrB*(Ts) *ΔtopA* strain. Figure 3E shows that it was indeed the case. Whereas the *recA* growth phenotype was dominant over the *recQ* one (Figure 3A), the reverse was observed for the *lexA3* allele i.e., the *gyrB*(Ts) *ΔtopA ΔrecQ lexA3* strain grew like the *gyrB*(Ts) *ΔtopA ΔrecQ* one (Figure 3E). Thus, these results indicate that the *lexA3* allele improved the growth of the *gyrB*(Ts) *ΔtopA* strain mostly by reducing the amount of RecA proteins, but at the same time that a minimal level of RecA-dependent recombination was required for the optimal growth of the *gyrB*(Ts) *ΔtopA* strain.

Our results showed that the RecF pathway for the loading of RecA on ssDNA was not involved in the RecA effects in the *gyrB*(Ts) *ΔtopA* strain (Figure 2). The RecBCD pathway is the other one involved in the loading of RecA on ssDNA in *E. coli*. The introduction of a *recB*::Tn*10* mutation in our *gyrB*(Ts) *ΔtopA* strain resulted in a strain that grew very poorly. Growth inhibition was clearly observed at 37°C, a temperature normally fully permissive for the growth of the *gyrB*(Ts) *ΔtopA* strain (Figure 3F, compare *gyrB*(Ts) *ΔtopA* and *gyrB*(Ts) *ΔtopA recB*). At 30°C, growth was barely detected (Figure 3F) and the strain did not show grow at 24°C even after 6 days of incubation. These results indicate that the RecA effects in the *gyrB*(Ts) *ΔtopA* strain are most likely mediated through the RecBCD pathway and, more importantly, that a RecA-independent RecB function is required for the survival of the *gyrB*(Ts) *ΔtopA* strain. Such a RecB function has been linked to replication fork regression that can occur when forks are stalled [53], [54]. Thus, these results may suggest that replication is problematic in the *gyrB*(Ts) *ΔtopA* strain. This is supported by the results presented below.

### An *oriC::aph* mutation still *oriC* competent in *topA* null but not in isogenic *topA*+ cells complements both the growth and chromosome segregation defects

Our data suggested that hypernegative supercoiling in *topA* null mutants triggered RecA-dependent recombination that led to the accumulation of RecQ-processed intermediates. Without a sufficient amount of type 1A topo activity to resolve these intermediates, chromosome segregation could not occur. However, how excess negative supercoiling stimulated RecA-dependent recombination to a level that caused chromosome segregation defects is unclear.

We have recently used a Tn*5* mutagenesis system to isolate genetic suppressors of the growth defect of a *gyrB*(Ts) *ΔtopA rnhA* strain (Materials and Methods; Usongo and Drolet, manuscript in preparation). The growth defect of this strain was previously shown to be related to chromosome segregation problems that could be corrected by overproducing topo III [17]. Improving gyrase activity also suppressed the chromosome segregation defects [17]. Moreover, our study of replication in this mutant led us to speculate that unregulated replication either from *oriC* or R-loops, or from both, could contribute to the segregation defects [34]. In agreement with this hypothesis, insertion mutants were found in loci involved in replication.

**Figure 4:**
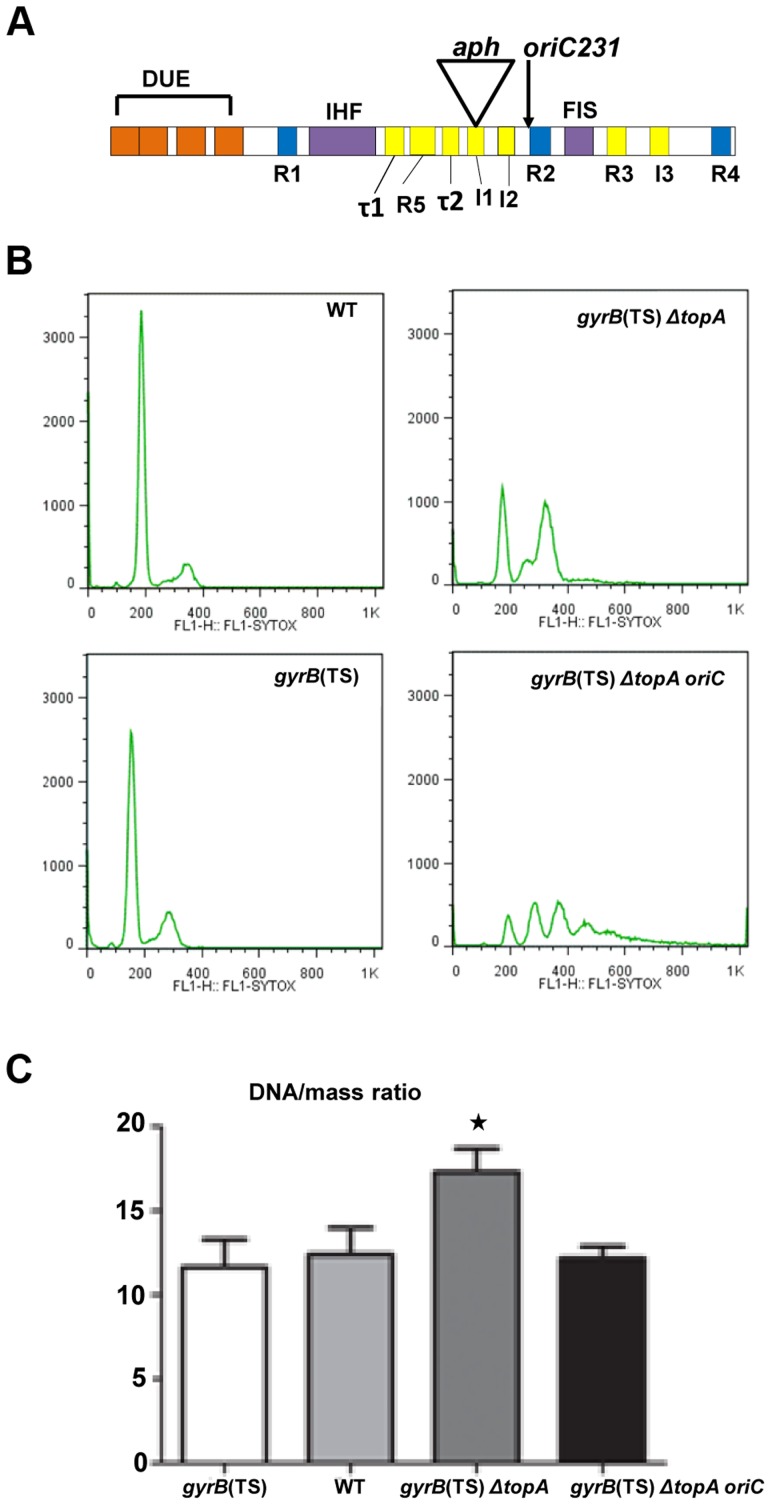
Replication initiation asynchrony and reduced DNA/mass ratio conferred by the *oriC15*::*aph* suppressor mutation. (a) Schematic representation of the minimal *oriC* region (245 bp) with its regulatory elements. DUE is the DNA unwinding element with its AT-cluster and 13-mer repeats L, M, and R (orange). DnaA binding sites: R1, R2 and R4 are high affinity sites (blue) whereas R3, R5, I1-3 and τ1-2 are low affinity sites (yellow). I1-3 and τ1-2 preferentially bind DnaA-ATP. IHF and FIS binding sites are also shown. For more details see [58]. *aph* indicates the insertion site of the *kan^r^* cassette in our *oriC15::aph* insertion mutant (position 142 in the 245 bp *oriC* region). The *oriC231* allele of Stepankiw *et al*. [57] spanning the left portion of *oriC* up to the arrow is shown for comparison (position 163 in the 245 bp *oriC* region). (b) Rifampicin run-out experiments for flow cytometry analysis were performed as described in Materials and Methods. Cells were grown in M9 minimal medium. (c) DNA/mass ratios were calculated as described in Material and Methods from three independent flow cytometry experiments. The strains used were: RFM443 (wild-type), RFM445 (*gyrB*(Ts)), RFM475 (*gyrB*(Ts) *ΔtopA*) and VU155 (RFM475 *oriC15::aph*). RFM475 has a significantly higher (*) DNA/mass ratio compared to RFM445 (p = 0.0199), RFM443 (p = 0.0274) and RFM475 *oriC* (p = 0.0292) Moreover, there is no statistical differences between the DNA/mass ratio of RFM475 *oriC* compared to RFM445 (p = 0.6587) and RFM443 (p = 0.8798).

In one mutant, the *kan^r^* cassette was found to be inserted within the *oriC* region, close to the middle (Figure 4A, *aph*). It was possible that the suppressed strain could survive without an active *oriC* region, as replication could occur from R-loops due to the absence of the *rnhA* gene (constitutive stable DNA replication, cSDR) [55]. Therefore, to verify if this *oriC15*::*aph* mutation was still competent for replication initiation, we tried to introduce it in wild-type (RFM443), *gyrB*(Ts) (RFM445) and *gyrB*(Ts) *ΔtopA* (RFM475) isogenic strains. Kanamycin resistant transductants were readily obtained for the *gyrB*(Ts) *ΔtopA* strain. Southern blot analysis confirmed that the *gyrB*(Ts) *ΔtopA* transductants carried the mutated but not the wild-type *oriC* region (Figure S4, RFM475 *oriC15*::*aph*). The few kanamycin resistant transductants of the wild-type and *gyrB*(Ts) strains were found to be false-positives as they kept the wild-type *oriC* region (Figure S4, a false positive RFM443 *kan^r^* is shown). Repeated transduction experiments yielded similar results. Therefore, we concluded that the *oriC15*::*aph* mutation was viable only when the *topA* gene was absent.

Our finding that overproducing RNase HI had no effect on the growth of the *gyrB*(Ts) *ΔtopA* strain carrying the *oriC15*::*aph* mutation (at 37 and 41°C, not shown), indicated that this strain does not replicate its chromosome via cSDR. This is in agreement with a previous report showing that, as opposed to an *rnhA* null mutant, a *topA* null mutant could not survive without a functional *oriC*/DnaA system [27]. Therefore, our *topA* null mutant most likely uses the *oriC15*::*aph* allele to initiate the replication of its chromosome. However, we can predict that this allele would be less active than a wild-type one and therefore should be able to complement the growth defect of a strain in which excess replication from *oriC* is growth inhibitory. The *dnaAcos* mutant, isolated as an intragenic suppressor of a *dnaA46* mutant, fails to grow at 30°C and below, because of excessive replication initiation from *oriC* [56]. A *dnaAcos* strain carrying the *oriC15*::*aph* mutation showed good growth at both 36 and 30°C, whereas an isogenic strain with a wild-type *oriC* region did not (Figure S5, *dnaAcos oriC15::aph* vs *dnaAcos*). Thus, this result confirmed that (i) the *oriC15*::*aph* mutation is functional in replication initiation and (ii) it is less active than a wild-type *oriC* region.

Our results with the *oriC15*::*aph* mutation suggested that topo I may play an important role in regulating replication initiation from *oriC*. In a previous study, the left-half of the *oriC* region was shown to be essential for *oriC* function *in vivo* [57]. This section carries the DUE (DNA unwinding element, AT-rich) region from which *oriC* duplex melting is initiated (Figure 4A) [58]. The smallest *oriC* fragment found to be functional *in vivo* was a fragment encompassing nucleotide 1 to 163 of the *oriC* region (Figure 4A, *oriC231*). However, a wild-type strain carrying this fragment was sensitive to rich media (LB). It was concluded that the right-half of *oriC* was essential for multi-forked replication that is required to support high growth rates in rich media [57]. Therefore, the fact that the *kan^r^* cassette was inserted at position 142 in the *oriC* sequence (Figure 4A), likely explains why our *oriC15*::*aph* mutation was not functional in a wild-type strain. However, not only was the mutation *oriC*-competent in our *topA* null mutant, it apparently allowed multi-forked replication, since our *gyrB*(Ts) *ΔtopA* strain was able to grow robustly in rich media. Therefore, these results suggest that topo I plays an important regulatory role at *oriC*.

Flow cytometry was used in rifampicin run-out experiments with cells grown in M9 medium at 37°C to investigate the regulation of replication initiation in our strains. As recently shown [34], both wild-type and *gyrB*(Ts) cells contained 2^n^ chromosome, thus indicating that replication initiation was well regulated in these strains (Figure 4B). Cells of the *gyrB*(Ts) *ΔtopA* strain had a near perfect 2^n^ chromosomal pattern with one small additional peak, showing some asynchrony (Figure 4B). However, flow cytometry analysis revealed that replication initiation was not well regulated in the *topA* null mutant carrying the *oriC15*::*aph* mutation, as peaks reflecting 1, 2, 3, or 4 chromosomes were clearly observed (Figure 4B, *gyrB*(Ts) *ΔtopA oriC*). Highly asynchronous replication was also previously detected in a wild-type strain carrying the *oriC231* mutation [57]. Flow cytometry analysis also revealed that the DNA/mass ratio was higher by roughly 40% in the *gyrB*(Ts) *ΔtopA* strain as compared to wild-type and *gyrB(Ts)* strains (Figure 4C). Introducing the *oriC15*::*aph* mutation into the *topA* null strain restored the DNA/mass ratio to the level seen in wild-type and *gyrB*(Ts) strains (Figure 4C, *gyrB*(Ts) *ΔtopA oriC*). Thus, the *oriC15*::*aph* mutation caused replication initiation to be less efficient in the *gyrB*(Ts) *ΔtopA* strain as shown by the loss of regulation and the lower DNA/mass ratio.

**Figure 5:**
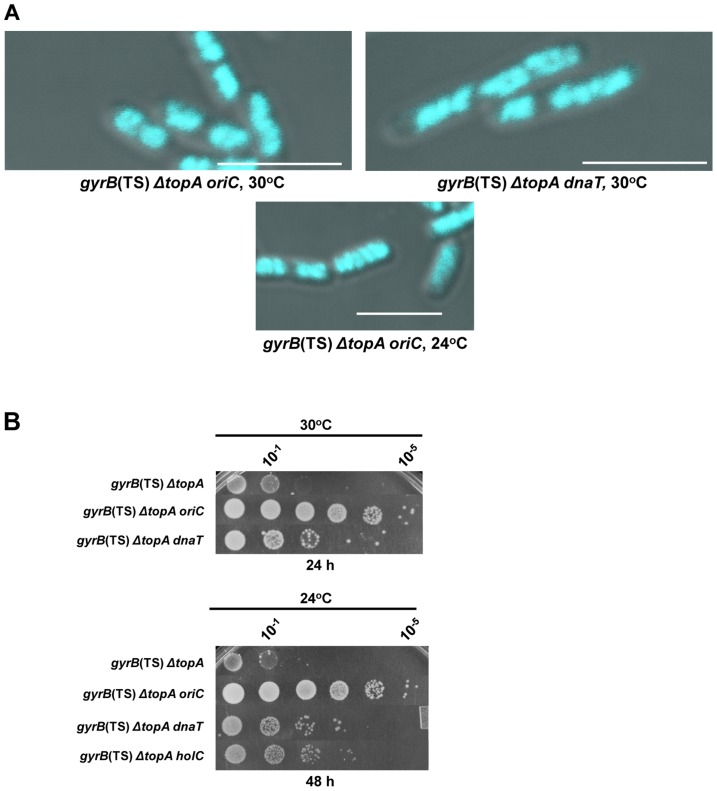
Effects of mutations affecting DNA replication on the growth and chromosome segregation defects in the *gyrB*(Ts) *ΔtopA* strain. (a) Representative superimposed images of DIC and fluorescence pictures of DAPI-stained cells grown at the indicated temperatures as described in Materials and Methods. Size bars are 5 µm. Additional images are shown in Figure S1 and S2. (b) Spot tests were performed at the indicated temperatures. The strains used are derivatives of RFM475 (*gyrB*(Ts) *ΔtopA*). They are: VU155 (RFM475 *oriC*), VU188 (RFM475 *dnaT*) and VU176 (RFM475 *holC2::aph*).

The *oriC15*::*aph* mutation was very effective in correcting the growth defect of our *gyrB*(Ts) *ΔtopA* strain at both 30 and 24°C (Figure 5B, compare *gyrB*(Ts) *ΔtopA* and *gyrB*(Ts) *ΔtopA oriC*). This mutation also significantly corrected the chromosome segregation defects of the *topA* null strain at both temperatures (Figure 5A, *gyrB*(Ts) *ΔtopA oriC*). Therefore, the *recA*-dependent chromosome segregation defects in the *topA* null mutant are likely related to excess replication from *oriC*. We conclude that one major role of *E. coli* topo I in genome maintenance is to prevent over-replication originating from *oriC*.

### Supercoiling- and R-loop-dependent growth and chromosome segregation defects in a *gyrB*(Ts) *ΔtopA ΔtopB* strain

We have recently shown that deleting *topA* could complement the growth defect of our *gyrB*(Ts) strain at non-permissive temperatures (40 to 42°C) by partially correcting its replication initiation and chromosome segregation defects [34]. However, we found that the *topB* gene was required for chromosome segregation and overproducing topo IV, the main cellular decatenase, could not substitute for *topB*. These results, and others, allowed us to conclude that topo III plays a role in replication that becomes essential when gyrase activity is defective. Here, we have confirmed that recombination was not involved by showing that deleting *recA* or *recQ* did not correct the growth and chromosome segregation defects of the *gyrB*(Ts) *ΔtopA ΔtopB* strain at a non-permissive temperature (40°C, Figure S6). Moreover, RNase HI overproduction had no effect. Thus, at non-permissive temperatures for the *gyrB*(Ts) allele, the growth and chromosome segregation defects of the *gyrB*(Ts) *ΔtopA ΔtopB* strain [34] are unrelated to recombination and R-loops.

**Figure 6:**
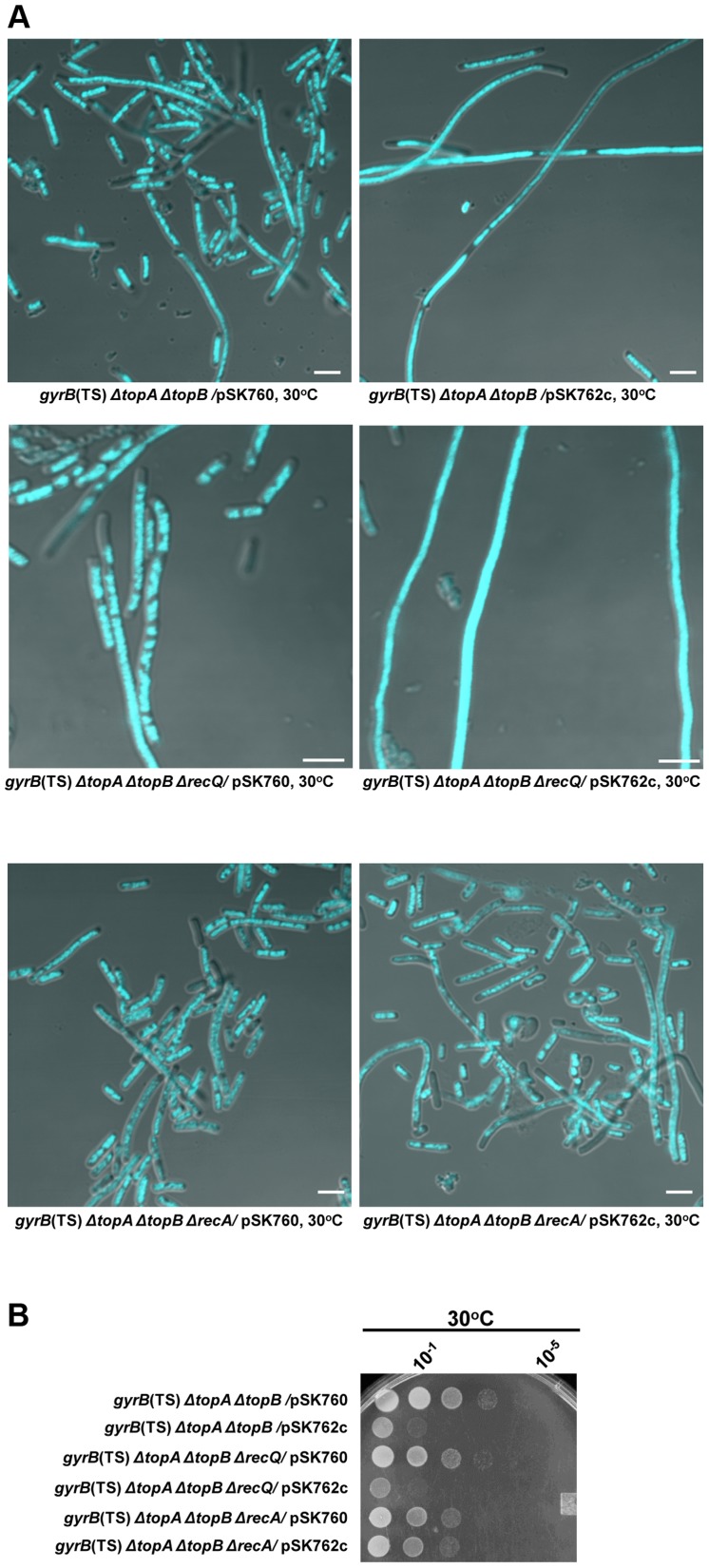
Effects of RNase HI overproduction and *recA* and *recQ* deletions on cells lacking type 1A topos. (a) Representative superimposed images of DIC and fluorescence pictures of DAPI-stained cells grown at 30°C as described in Materials and Methods. Size bars are 5 µm. (b) Spot tests at 30°C. The LB plate was incubated for 24 h. The strains used are all derivative of RFM475 (*gyrB*(Ts) *ΔtopA*). They are: VU306 (RFM475 *ΔtopB*/pSK760), VU333 (RFM475 *ΔtopB*/pSK762c), VU363 (RFM475 *ΔtopB ΔrecQ*/pSK760), VU365 (RFM475 *ΔtopB ΔrecQ*/pSK762c), VU375 (RFM475 *ΔtopB ΔrecA*/pSK760) and VU379 (RFM475 *ΔtopB ΔrecA*/pSK762c). pSK760 carries the *rnhA* gene for RNase HI overproduction, whereas pSK762c carries a mutated and inactive *rnhA* gene.

It was observed that the optimal temperature for the growth of the *gyrB*(Ts) *ΔtopA ΔtopB* strain was 37°C. Indeed, at 30°C the growth defect was found to be exacerbated (Figure S7a, compare 37 and 30°C for *gyrB*(Ts) *ΔtopA ΔtopB*/pSK762c). This strain also generated a higher proportion of longer cells at 30 than 37°C (Figure S7b, *gyrB*(Ts) *ΔtopA ΔtopB*, 37 vs 30°C). Since gyrase was re-activated at 30°C, we considered the possibility that deleting *topB* exacerbated *topA* phenotypes at this temperature. If this was true, overproducing RNase HI should have a positive effect on growth and chromosome segregation in our triple mutant. Indeed, this turned out to be true as the spot assay revealed that growth was better, by at least two logs, when RNase HI was overproduced (Figure 6b, compare *gyrB*(Ts) *ΔtopA ΔtopB*/pSK760, RNase HI overproduced and *gyrB*(Ts) *ΔtopA ΔtopB*/pSK762c, RNase HI not overproduced). Moreover, the strong chromosome segregation defects illustrated by the formation of very long filaments fully packed with diffuse DNA, were significantly corrected by overproducing RNase H. In this case, cells were shorter and the DNA was more compact (Figure 6A). Thus, R-loops-related problems of a *topA* null mutant were exacerbated by deleting *topB* and were mostly expressed as chromosome segregation defects.

### RecA-dependent but RecQ-independent growth and chromosome segregation defects in a *gyrB*(Ts) *ΔtopA ΔtopB* strain at 30°C

The deletion of *recA* significantly improved the growth of the *gyrB*(Ts) *ΔtopA ΔtopB* strain at 30°C, though this was not as effective as overproducing RNase HI (Figure 6B, compare *gyrB*(Ts) *ΔtopA ΔtopB ΔrecA*/pSK762c and *gyrB*(Ts) *ΔtopA ΔtopB*/pSK760). However, deleting *recA* was at least as effective as overproducing RNase HI in correcting the chromosome segregation defects of the *gyrB*(Ts) *ΔtopA ΔtopB* strain (Figure 6A, compare *gyrB*(Ts) *ΔtopA ΔtopB ΔrecA*/pSK762c and *gyrB*(Ts) *ΔtopA ΔtopB*/pSK760). Furthermore, overproducing RNase HI had no effects on growth and chromosome segregation when *recA* was deleted (Figure 6A, compare *gyrB*(Ts) *ΔtopA ΔtopB ΔrecA*/pSK760 and *gyrB*(Ts) *ΔtopA ΔtopB ΔrecA*/pSK762c). These results demonstrate that the R-loop-dependent chromosome segregation defects in cells lacking type 1A topos, are also dependent on RecA.

Unlike inactivating *recA*, the deletion of *recQ* did not correct the phenotypes of the *gyrB*(Ts) *ΔtopA ΔtopB* strain (Figure 6, compare *gyrB*(Ts) *ΔtopA ΔtopB ΔrecQ*/pSK762c and *gyrB*(Ts) *ΔtopA ΔtopB*/pSK762c). However, RNase HI overproduction was still able to correct these phenotypes when *recQ* was absent (compare *gyrB*(Ts) *ΔtopA ΔtopB ΔrecQ*/pSK760 and *gyrB*(Ts) *ΔtopA ΔtopB ΔrecQ*/pSK762c). Thus, the RecA- and R-loop-dependent growth and chromosome segregation defects of the *gyrB*(Ts) *ΔtopA ΔtopB* strain are not caused by the accumulation of RecQ-processed recombination intermediates that are substrates for type 1A topos. As RecA was previously shown to be required for cSDR that initiates from R-loops [55], over-replication could possibly be the triggering event for the growth and chromosome segregation defects of cells lacking type 1A topos. This is supported by the genetic evidence presented below.

### Suppressor mutations affecting R-loop- and/or *oriC*-dependent replication significantly correct the growth and chromosome segregation defects in *gyrB*(Ts) *ΔtopB topA20*::Tn*10* strains at 30°C

**Figure 7:**
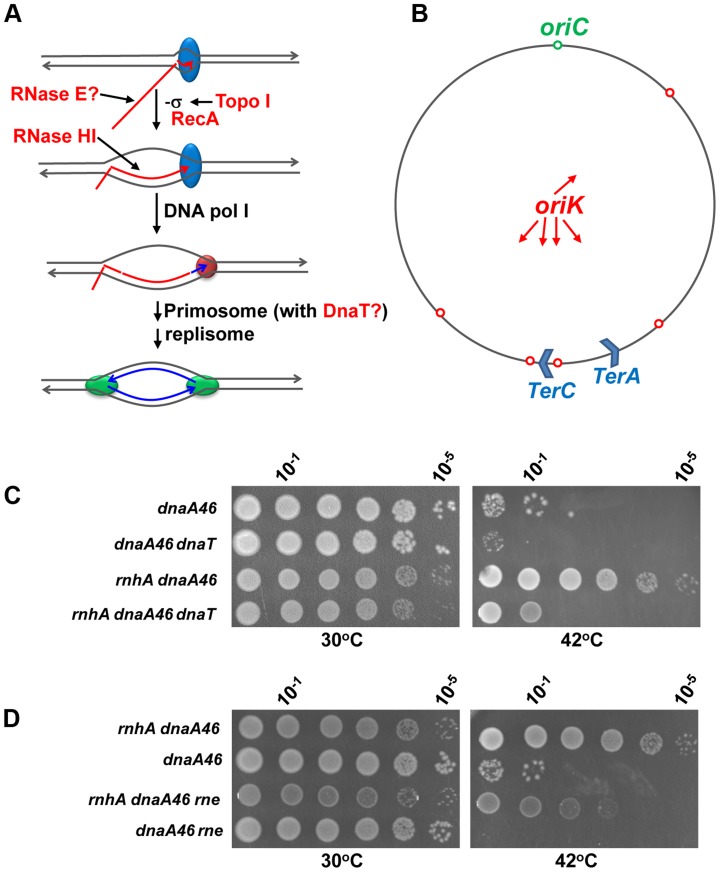
The *dnaT18*::*aph* and *rne59*::*aph* suppressor mutations inhibit cSDR in an *rnhA* strain. (a) Model for constitutive stable DNA replication (cSDR) [55], [61]. R-loop forms during transcription when the nascent RNA hybridizes with the template DNA strand behind the moving RNA polymerase. Both transcription-induced negative supercoiling and RecA protein promote R-loop formation. DNA pol I synthesizes DNA from the 3′ end of the hybridized RNA for primosome (PriA-dependent) assembly. Eventually, the primosome allows the assembly of two replisomes for bidirectional replication. The proteins that are included in the present study are shown in red: topo I relaxes transcription-induced negative supercoiling; RecA promotes the hybridization of the template DNA strand with the nascent RNA [86], [87]; RNase HI degrades the RNA of the R-loop; RNase E may inhibit R-loop formation by degrading the nascent RNA; DnaT may play a role in cSDR via the primosome. (b) A map of the *E. coli* chromosome showing the normal origin of replication (*oriC*), the putative cSDR origins of replication (*oriK*, [55]) and two of the ten *ter* sites, with *terC* believed to be a site where many convergent replication forks meet [88]. (c) and (d). Spot tests. The LB plates were incubated for 24 h, at 30 or 42°C as indicated. The strains used were: MD48 (*dnaA46*(Ts)), JE35 (*rnhA dnaA46*(Ts)), VU204 (*dnaA46*(Ts), *dnaT*), VU200 (*rnhA dnaA46*(Ts) *dnaT*), JE36 (*rnhA dnaA46*(Ts) *rne*) and JE119 (*dnaA46*(Ts) *rne*). At 42°C, the few colonies of strain MD48 (at 10^0^ and 10^−1^) were made of cells that have acquired compensatory mutations, as they grew robustly upon restreaking them at the same temperature.

One of the best suppressors of the growth defect of the *gyrB*(Ts) *ΔtopA rnhA* strain that displays cell filamentation and chromosome segregation phenotypes similar to our *gyrB*(Ts) *ΔtopA ΔtopB* strain, had the *kan^r^* cassette inserted within the promoter region of the *dnaT* gene (Figure S8A). DnaT is one of the various proteins that constitute the primosome (including PriA [59]). This protein complex allows the assembly of a replisome outside of *oriC*. Interestingly, the first mutation found to inhibit SDR mapped within *dnaT* [60]. The SOS-dependent form of stable DNA replication (iSDR) was shown to be inhibited in this case [55]. However, the involvement of *dnaT* in the R-loop-dependent form of SDR (cSDR) is still unknown [61]. To test this, the *dnaT18*::*aph* mutation was introduced in a *dnaA46*(Ts) strain also carrying an *rnhA* null mutation. The absence of RNase HI allows the *dnaA46*(Ts) strain to grow at 42°C as it can replicate its chromosome from R-loops (Figure 7A and B). Therefore, the fact that the *dnaT18*::*aph* allele inhibited the growth of the *dnaA46*(Ts) *rnhA* strain at 42°C, indicated that the *dnaT* gene was required for cSDR (Figure 7C, 42°C, compare *rnhA dnaA46* and *rnhA dnaA46 dnaT*).

**Figure 8:**
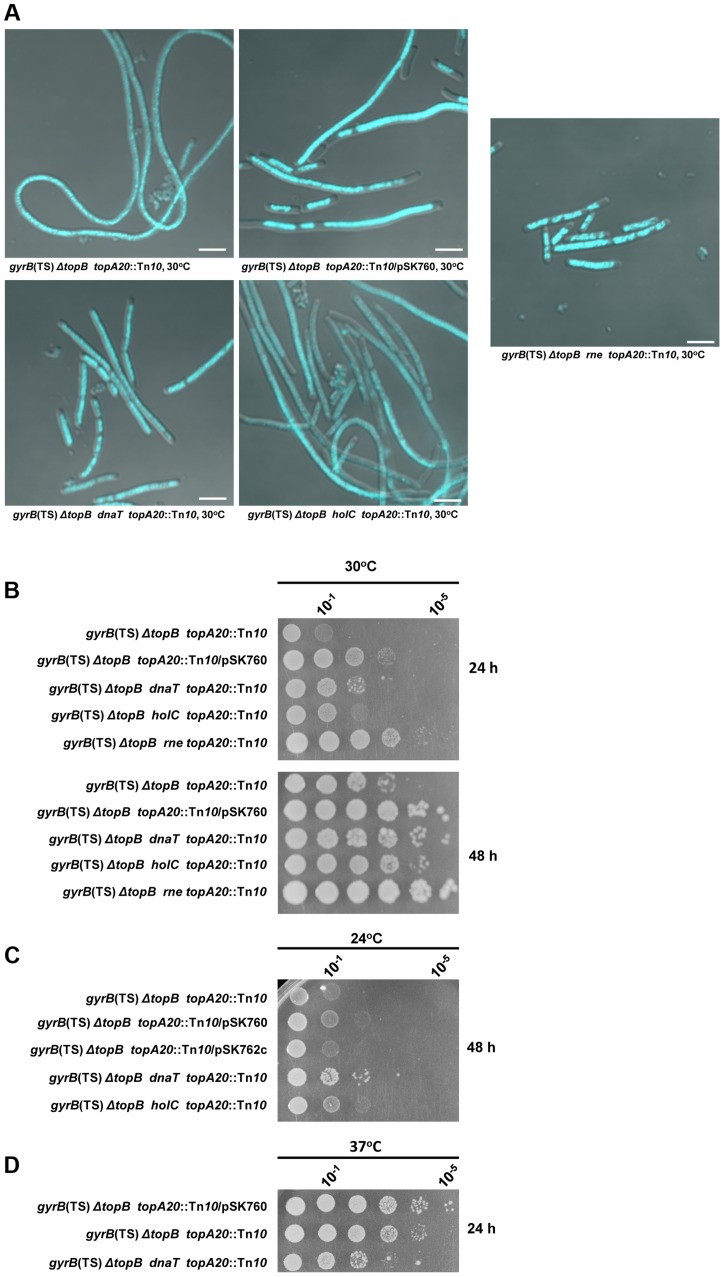
Effects of *dnaT18*::*aph*, *rne59*::*aph* and *holC2*::*aph* suppressor mutations on cells lacking type 1A topos. Representative superimposed images of DIC and fluorescence pictures of DAPI-stained cells grown at 30°C as described in Materials and Methods. Size bars are 5 µm (a). Spot tests at 30°C (b), 24°C (c) and 37°C (d). The LB plates were incubated for the indicated times. The strains used are all derivative RFM445 *ΔtopB* (strain VU409: *gyrB*(Ts), *ΔtopB*). They are: VU421 (VU409 *topA20*::Tn*10*), VU422 (VU409 *topA20*::Tn*10*/pSK760), VU425 (VU409 *topA20*::Tn*10*/pSK762c), VU441 (VU409 *dnaT topA20*::Tn*10*), VU469 (VU409 *holC topA20*::Tn*10*), VU473 (VU409 *rne topA20*::Tn*10*). pSK760 carries the *rnhA* gene for RNase HI overproduction, whereas pSK762c carries a mutated and inactive *rnhA gene*.

The *dnaT18*::*aph* mutation was also found to partially correct the chromosome segregation defects of the *gyrB*(Ts) *ΔtopA rnhA* strain (Figure S9). This suggested that replication from R-loops could, at least in part, be responsible for these problems. We therefore tested the ability of the *dnaT18*::*aph* mutation to correct similar defects in cells lacking type 1A topos. For this purpose, a different null allele of *topA*, the *topA20*::Tn*10* allele that was previously shown to behave similarly to the *ΔtopA* allele used in the present study, was chosen [11]. A *ΔtopB gyrB*(Ts) strain was used in which the *topA20*::Tn*10* allele was either immediately introduced to obtain the *ΔtopB gyrB*(Ts) *topA20*::Tn*10* control strain, or introduced after the *dnaT18*::*aph* allele to obtain the *ΔtopB gyrB*(Ts) *dnaT18*::*aph topA20*::Tn*10* strain. The chromosome segregation defects were found to be more severe in our new *ΔtopB gyrB*(Ts) *topA20*::Tn*10* strain as compared to the other one carrying the *ΔtopA* allele (compare Figure 6A, *gyrB*(Ts) *ΔtopA ΔtopB*/pSK762c and Figure 8A, *ΔtopB gyrB*(Ts) *topA20*::Tn*10* and data not shown). Indeed, the *ΔtopB gyrB*(Ts) *topA20*::Tn*10* strain at 30°C produced almost exclusively extremely long filaments that were fully packed with diffuse DNA. This could be related to our previous observation that R-loop-related problems in the absence of topo I were more severe in strains carrying the *topA20*::Tn*10* allele instead of the *ΔtopA* one [62]. RNase HI overproduction also significantly corrected both the growth and chromosome segregation defects of our *ΔtopB gyrB*(Ts) *topA20*::Tn*10* strain (Figure 8A and B, compare *ΔtopB gyrB*(Ts) *topA20*::Tn*10* and *ΔtopB gyrB*(Ts) *topA20*::Tn*10*/pSK760). However, at 24°C, RNase HI overproduction had no effect (Figure 8C, compare *ΔtopB gyrB*(Ts) *topA20*::Tn*10*/pSK760 and *ΔtopB gyrB*(Ts) *topA20*::Tn*10*/pSK762c). This was expected, as the cold-sensitivity of cells lacking topo I is not corrected by RNase HI overproduction (see above).

It was found that the *dnaT18*::*aph* mutation was at least as effective as RNase HI overproduction in correcting the chromosome segregation defects of the *ΔtopB gyrB*(Ts) *topA20*::Tn*10* strain (Figure 8A, compare *ΔtopB gyrB*(Ts) *dnaT topA20*::Tn*10*, *ΔtopB gyrB*(Ts) *topA20*::Tn*10*/pSK762c and *ΔtopB gyrB*(Ts) *topA20*::Tn*10*/pSK760). However, RNase HI overproduction was slightly better than the *dnaT18*::*aph* mutation in correcting the growth defect (Figure 8B, compare *ΔtopB gyrB*(Ts) *topA20*::Tn*10*/pSK760 and *ΔtopB gyrB*(Ts) *dnaT topA20*::Tn*10*). The *dnaT18*::*aph* also had a negative effect on the growth of the *ΔtopB gyrB*(Ts) *topA20*::Tn*10* strain at 37°C (Figure 8D, compare *ΔtopB gyrB*(Ts) *dnaT topA20*::Tn*10* and *ΔtopB gyrB*(Ts) *topA20*::Tn*10*). This could be due to the presence of the *gyrB*(Ts) allele that was previously shown, at this semi-permissive temperature, to be incompatible with a mutation (*priA* null) inactivating the primosome [63]. Thus, our results support the hypothesis that the R-loop and RecA-dependent chromosome segregation defects in cells lacking type 1A topos are, at least in part, related to over-replication initiated from R-loops. The fact that the *dnaT18*::*aph* mutation slightly promoted the growth of our *topA* null mutant (Figure 5B, 30 and 24°C, compare *gyrB*(Ts) *ΔtopA dnaT* vs *gyrB*(Ts) *ΔtopA*), suggests that cSDR is primarily a problem for *topA* null cells that is exacerbated by deleting *topB*. This would be consistent with the assumption that topo I is the primary type 1A topo involved in the inhibition of R-loop formation [47].

Seven different *kan^r^* insertion mutations in the C-terminal region of RNase E, the main endoribonuclease in *E. coli* (Usongo and Drolet, manuscript in preparation), were found to suppress the growth defect of our *topA rnhA gyrB*(Ts) strain. Interestingly, experimental evidence for an interplay between RNase HI and RNase E in RNA degradation has been reported [64], [65]. One of these *rne* mutations (*rne59::aph*, Figure S8C) was introduced in a *dnaA46*(Ts) *rnhA* strain to test its effect on cSDR. The presence of the *rne59::aph* mutation significantly reduced the ability of the *dnaA46*(Ts) *rnhA* strain to grow at 42°C (by 2 to 3 logs; Figure 7D, 42°C, *rnhA dnaA46* vs *rnhA dnaA46 rne*). This result shows that the mutated RNase E inhibited cSDR.

A *topA topB gyrB*(Ts) strain was constructed, with the *topA20*::Tn*10* allele as described above, that carried the *rne59::aph* mutation. The *rne59::aph* mutation was found to be slightly better than RNAse HI overproduction to correct the growth defect of cells lacking type 1A topos (Figure 8B, compare *ΔtopB gyrB*(Ts) *rne topA20*::Tn*10* and *ΔtopB gyrB*(Ts) *topA20*::Tn*10*/pSK760). Furthermore, it was at least as effective as RNase HI overproduction and the *dnaT18*::*aph* mutation to correct the chromosome segregation defects in these cells (Figure 8A). Thus, our results with the *rne59::aph* mutation lend further support to the hypothesis that cells lacking type 1A topos suffer from excess replication originating from R-loops.

The origins of replication for cSDR (*oriK*s) in *rnhA* null mutants are mostly found within or close to the *ter* region where bi-directional replication initiated at *oriC* normally terminates [55]. Thus, the origin to terminus (*oriC/ter*) ratio, is expected to be lowered by the occurrence of cSDR. This is indeed what was found for the *rnhA* null mutant (Figure S10, RFM443 vs RFM430 *rnhA::cam*). The *ori/ter* ratio was also similarly reduced in the *topA* null mutant, thus supporting the occurrence of cSDR in the absence of topo I (Figure S10, RFM475).

Several of our *kan^r^* insertion mutants were found to reduce the expression of the *holC* gene (Usongo and Drolet, manuscript in preparation). In a previous study, *kan^r^* insertion mutants that reduced the expression of the *holC* gene were also found to suppress the growth defect of a *dnaAcos* strain [66]. The *holC* gene encodes the χ subunit of DNA pol III, the replicative polymerase in *E. coli* [67]. The χ subunit interacts with SSB and this interaction was recently shown to play an important role in replisome establishment and maintenance [68]. The *holC2*::*aph* mutation was tested for its ability to suppress phenotypes of cells lacking type 1A topos. For this purpose, a *topA topB gyrB*(Ts) *holC2*::*aph* strain, carrying the *topA20*::Tn*10* allele, was constructed. The *holC2*::*aph* mutation was shown to slightly correct the growth defect of cells lacking type 1A topos activity (Figure 8B and C, 30 and 24°C respectively, *ΔtopB gyrB*(Ts) *holC topA20*::Tn*10* vs *ΔtopB gyrB*(Ts) *topA20*::Tn*10*). Both cell length and the amount of DNA were also slightly reduced (Figure 8A). The fact that *holC* mutations by themselves can cause filamentation and chromosome segregation defects [68], may explain why the *holC2*::*aph* mutation only partially corrected the phenotypes of the *ΔtopB gyrB*(Ts) *topA20*::Tn*10* strain.

The *holC2*::*aph* mutation also partially corrected the growth defect of our *topA* null mutant (Figure 5B, 24°C, 48 h; compare *gyrB*(Ts) *ΔtopA* and *gyrB*(Ts) *ΔtopA holC*). Moreover, in rifampicin run-out experiments, replication did not appear to be well regulated in the *topA* null mutant carrying the *holC2*::*aph* mutation, as peaks reflecting 1, 2, 3, or 4 chromosomes were clearly observed (Figure S11, compare *gyrB*(Ts) *ΔtopA* and *gyrB*(Ts) *ΔtopA holC*). This result supports the hypothesis that the χ subunit of pol III plays a role in replication initiation [68] and therefore suggests that initiation from *oriC* could also be problematic in cells lacking both type 1A topos.

## Discussion

The work described in this manuscript, which focused on the role of type 1A topos in genome maintenance in *E. coli*, revealed new important functions in replication and chromosome segregation for both topo I and III. Before this study, not much was known about the role of these enzymes in genome maintenance as opposed to the situation for eukaryotic type 1A topos. It was generally believed that topo I and III have distinct cellular functions, with topo I regulating supercoiling and R-loop formation and topo III being involved in chromosome segregation during replication. Here, our results show that *E. coli* type 1A topos play major roles in genome maintenance by inhibiting inappropriate replication and, as is the case for eukaryotic type 1A enzymes, by acting with RecQ to resolve RecA-generated recombination intermediates. Furthermore, while inhibiting replication from *oriC* is specific to topo I that regulates supercoiling, both the prevention of over-replication from R-loops and the activity with RecQ appear to be shared by both type 1A topos. This may suggest that these two functions are the major ones for type 1A topos and that they may have been conserved throughout evolution. Below, we discuss about the newly identified functions of *E. coli* type 1A topos in this work.

### *E. coli* type 1A topos and RecQ

As stated in the introduction, the strand passage activity of *E. coli* topo III, but not topo I, was shown to be strongly stimulated by RecQ *in vitro* [35]–[37]. This would suggest that *E. coli* topo III and RecQ can act together to maintain the stability of the genome, as shown in eukaryotic cells [69]. However, no clear evidence for such a role of topo III has been reported in *E. coli*. Recent experimental evidence points to a role for topo III in chromosome segregation related to replication and independent of RecQ ([33], [34]; this work). In fact, the data presented here suggest that topo I, not topo III, is the primary type 1A topo acting with RecQ in *E. coli*. Indeed, the strong chromosome segregation and growth defects of *topA* null cells at low temperatures were shown to be partially corrected by deleting *recQ* or *recA*, independent of the RecFOR pathway and by overproducing topo III, a protein that is normally of very low abundance. Moreover, both deleting *recQ* and overproducing topo III were found to be epistatic to *recA* in correcting the growth problems. This is consistent with RecQ processing RecA-dependent recombination intermediates in such a way that they can only be resolved by a type 1A topo, as is the case in eukaryotic cells. In this context, topo III overproduction would substitute for topo I and perform the resolution, thus meaning that topo III can also perform this reaction *in vivo*.

Alternatively, in the absence of *topA*, DNA substrates for topo I may accumulate and some of them could be processed by topo III, thus leading to the depletion of this very low abundant protein. This situation would lead to the accumulation of RecQ-processed recombination intermediates, if topo III normally resolves them. However, we think that this is unlikely because while a *topA recQ* strain grow very well, deleting *topB* make this strain very sick with phenotypes identical to those of *topA topB* null cells. If *recQ* was acting with *topB*, then deleting *topB* should have had no effect on the growth of the *topA recQ* strain. Altogether, our results are more consistent with topo I being the primary type 1A topo working with RecQ in *E. coli*.

Despite the previously observed lack of stimulation of topo I activity by RecQ *in vitro*, we still believe that these two proteins can functionally interact. Indeed, it may be that the optimal experimental conditions and/or the appropriate substrate for their functional interaction have not yet been well defined. Alternatively or additionally, the much higher abundance of topo I *in vivo* as compared to topo III may compensate for its lower level of activity with RecQ. In fact, the finding that either *E. coli* topo I expression or a *SGS1* mutation could compensate for the absence of Top3 in *S. cerevisiae* [38]–[40], supports the assumption that *E. coli* topo I can act with RecQ *in vivo*. Moreover, in an *in vitro* system for DHJs resolution by BLM helicase with a type 1A topo, *E. coli* topo I was shown to efficiently substitute for human topo IIIα [70]. Hsieh and co-workers have recently obtained experimental evidence for their “unravel and unlink” model whereby BLM first melts a DNA region to which RPA protein binds and topo IIIα acts to resolve a DHJ [1], [43]. Indeed, a topo IIIα mutant unable to physically interact with BLM was shown to partially resolve a DHJ in the presence of RPA, thus suggesting that the functions of the two proteins may be separated [43]. A similar model might also be proposed for RecQ acting with topo I, the activity of which can be stimulated by SSB [71], as the two proteins do not physically interact.

Interestingly, whereas topo I is present in all bacteria, topo III is present only in a few bacteria [72]. Therefore, if a collaboration between a type 1A topo and a RecQ-like helicase is also required in bacteria, it is not surprising that topo I performs this function. However, why such a function would be required in bacteria is currently unknown. In diploid organisms RecQ-like helicases act in concert with topo III to prevent the exchange of genetic material between DNA molecules involved in recombination (DHJ dissolution; [73]).

In the present study, it was found that inactivating *recB* almost completely inhibited the growth of our *gyrB*(Ts) *ΔtopA* mutant, while deleting *recA* improved its fitness. This indicated that a RecA-independent RecB function was required for the survival of the *gyrB*(Ts) *ΔtopA* strain. Such a RecB function has been linked to replication forks regression that can occur when forks are stalled [53], [54]. In the cell, RecB is present in the RecBCD complex that has both a recombination (RecA loading at χ sites) and a dsDNA degradation (exonuclease V) function. It has been hypothesized that upon fork reversal, a HJ forms and degradation of the dsDNA end is initiated by RecBCD. Following the encounter of a χ site and in the presence of RecA, homologous recombination can take place, leading to the formation of a second HJ. The involvement of homologous recombination is supported by the observation that, as opposed to *recB* mutants, *recD* mutants, that lack the dsDNA degradation activity of RecBCD, can survive under conditions that promote extensive replication forks reversal if the *recA* gene is present [74], [75]. Next, the two HJs can be resolved by RuvABC. Alternatively, as is the case during the process of genetic exchange in diploid organisms, we propose that RecQ can act on the two HJs to promote convergent branch migration. This would lead to the formation of a hemicatenane that must be unlinked by a type 1A topo to allow chromosome segregation. In the absence of RecA, the second HJ does not form and the dsDNA is instead degraded by the RecBCD complex up to the first HJ to produce a fork structure that is used to restart replication. In this context, RecQ does not promote the formation of hemicatenanes and, as a result, chromosomes segregation is not impeded by the lack of type 1A topos activity.

Experimental evidence for replication forks reversal has been reported in *E. coli* cells carrying defective DNA helicases involved in replication (DnaB and Rep; [53], [74]) and, more recently, following replication-transcription collisions [75]. We speculate that the high level of negative supercoiling in the *gyrB*(Ts) *ΔtopA* strain at low temperatures promotes the formation of alternative non-B DNA structures that may cause the stalling of replication forks and their reversal. Alternatively, such non-B DNA structures may first block transcription and the arrested RNA polymerases may, in turn, stop the progression of replication forks to cause their reversal. Furthermore, over-replication that occurs in this strain likely exacerbates the problem and makes the cell unable to adequately deal with the reversed forks. Clearly, more work will be required to fully characterize the role(s) of type 1A topos acting with RecQ in bacteria and to find out under which circumstances this activity would be required in various DNA transactions.

### *E. coli* type 1A topos in replication

In *E. coli*, replication initiated at *oriC* is tightly regulated so that it occurs once and only once per cell cycle [58]. This process is synchronized with the “initiation mass”. DNA supercoiling is among the many elements, including DnaA that are required for replication initiation at *oriC*. Indeed, *in vitro* replication initiation necessitates that the *oriC* plasmid be negatively supercoiled [76]. *In vivo*, deleting *topA* was found to correct the thermo-sensitive growth of a *dnaA*(Ts) mutant [27] and altering gyrase supercoiling activity inhibited replication initiation from *oriC* [77]. Moreover, we have recently shown that a *topA* deletion could correct the replication initiation defect of a strain defective for gyrase supercoiling activity [34]. Interestingly, in a screen to isolate DnaA inhibitors a compound was recently found to rescue a *dnaAcos* mutant from lethal hyperinitiation by targeting gyrase [78]. Thus, *in vitro* and *in vivo* data demonstrate that negative DNA supercoiling is required for replication initiation from *oriC*.

The recent determination of the crystal structure of a truncated DnaA ortholog in complex with ssDNA supports a model whereby DnaA opens the *oriC* region by a direct ATP-dependent stretching mechanism [79]. This work provides the strongest evidence to date for a direct participation of DnaA in DNA melting at *oriC*, and is fully compatible with other elements, such as DNA supercoiling, also playing a role in this process. In a recent biochemical study, DNA fragments containing at least the left portion of *oriC* up to I1 or I2 (Figure 4a) were shown to be required for DnaA-ATP binding to ssDUE in the absence of torsional stress [80]. This result is totally consistent with our finding that an *oriC* region lacking these I1 and I2 sequences (*oriC15*::*aph*) is functional in a *topA* null mutant, where the negative supercoiling level is elevated, but not functional in an isogenic *topA^+^* strain. Thus, our results, together with those reported in the two studies mentioned above, suggest that DNA supercoiling plays an important regulatory role at *oriC*.

When *topB* was deleted from a *topA* null mutant, a new growth inhibitory phenotype, again related to replication, appeared at temperatures where the *oriC*-related phenotype was attenuated. Our data suggest that this major phenotype in the absence of type 1A topos is related to replication from R-loops (cSDR). This is consistent with a major role of topo I in the inhibition of R-loop formation and with the identification of topo I, like RNase HI [81], as a specificity factor to inhibit replication initiation at sites other than *oriC* (e.g. R-loops), in an *in vitro* system [28]. Thus, although the strong phenotype expressed such as extensive cell filamentation, unsegregated nucleoids and growth inhibition, is triggered by deleting *topB*, cSDR is probably also activated in our single *topA* mutant. This is supported by the fact that the *dnaT18*::*aph* mutation improved the growth of our *topA* mutant and by the finding that, as was the case in an *rnhA* null mutant, the *ori/ter* ratio was lower in this *topA* mutant as compared to a wild-type strain. However, even if cSDR is activated in *topA* null mutants, the *oriC*/DnaA system is still required in these cells to replicate the chromosome. A similar situation has been described for *recG* mutants, in which cSDR is also activated but cannot support replication of the whole chromosome [82], [83].

As the strong phenotype is due to the simultaneous absence of both type 1A topos, it is likely related to similar functions performed by the two enzymes. We have previously shown that an R-loop was a hot-spot for topo III activity *in vitro* [47]. By acting on an R-looped plasmid, topo III was shown to destabilize the R-loop. As topo III can travel with the replication fork [35], it could possibly act by destabilizing R-loops blocking the progression of the replication forks. Interestingly, topo III was recently shown to prevent R-loop accumulation during transcription in mammalian cells [84]. Thus, inhibition of R-loop formation might be another important function of type 1A topos that has been conserved throughout evolution. The absence of a type 1A topo activity for decatenation (e.g. RecQ with topo I) also likely contributes to the strong chromosome segregation defects seen in cells lacking both topo I and III.

## Materials and Methods

### Bacterial strains and plasmids

Bacterial strains used in this study are all derivatives of *E. coli* K12 and are listed in Table S1. Details on their constructions as well as the list of plasmids used in this study are also given in Table S1. Transductions with P1*vir* were performed as described previously [34]. PCR was used to confirm that the expected gene transfer occurred in the selected transductants.

### Insertional mutagenesis

Insertional mutagenesis with pRL27 was performed in a *topA rnhA gyrB*(Ts) strain and will be described in details elsewhere (Usongo and Drolet, manuscript in preparation). Briefly, pRL27 carries a hyperactive Tn*5* transposase gene under the control of the *tetA* promoter, and an insertional cassette with a kanamycin resistance gene (*aph*) and a *pir*-dependent origin (*oriR6K*) bracketed by Tn5 inverted repeats [85]. Following electroporation of pRL27 in a *pir-* background, the *kan^r^* cassette inserts randomly into the chromosome. A *topA rnhA gyrB*(Ts)/pBAD18*rnhA* strain was electroporated with pRL27 and plated on LB containing 25 µg/ml kanamycin at 40°C, to select for suppressors that grew in the absence of arabinose (no RNase HI produced). At this temperature, the strain does not grow because of extensive inhibition of the supercoiling activity of gyrase [17], combined with over-replication ([34] and see below). P1*vir* was grown on the *kan^r^* clones that re-grew at 40°C and each phage lysate was used to infect a *topA rnhA gyrB*(Ts)/pPH1243 strain, that normally grows only in the presence of IPTG, to overproduce topo III from pPH1243 [17]. Transductants were selected on LB plates containing IPTG and kanamycin (50 µg/ml) at 37°C. Transductants that re-grew in the absence of IPTG were selected for further characterization. Four of the insertion mutants, described in Figure 4 and S8, were used in the present study.

### Spot tests

Cells from glycerol stocks were resuspended in LB to obtain an OD_600_ of 0.6. Five µl of 10-fold serial dilutions were then spotted on LB plates that were incubated at the indicated temperatures. The experiments were performed with cells from glycerol stocks to minimize the chance of selecting cells with compensatory mutations. However, we eventually found that similar results were obtained whether the cells were from glycerol stocks or from overnight liquid cultures (not shown).

### Microscopy

Cells were grown overnight at 37°C in liquid LB medium supplemented with the appropriate antibiotics. Overnight cultures were diluted in LB medium to obtain an OD_600_ of 0.01 and grown at the indicated temperature to an OD_600_ of 0.8. The cells were recovered and prepared for microscopy as previously described [17]. Pictures (fluorescence (DAPI) and DIC) were randomly taken with a LSM 510 Meta confocal microscope from Zeiss. The images were processed using Adobe Photoshop. Representative images are shown both in the Results and Supporting Information sections.

### Flow cytometry

The procedure for flow cytometry in rifampicin run-out experiments with cells grown in M9 medium has been described [34]. The DNA/mass ratio was calculated has previously reported [34].

## Supporting Information

Figure S1 Chromosome segregation defects in a *ΔtopA gyrB*(Ts) strain at 30°C. Superimposed images of DIC and fluorescence pictures of DAPI-stained cells grown at 30°C, unless otherwise indicated, as described in Materials and Methods. Size bars are 5 µm. The strains used are all derivatives of RFM475 (*gyrB*(Ts) *ΔtopA*) except RFM445 (*gyrB*(Ts)). They are: VU287 (RFM475/pSK760), VU155 (RFM475 *oriC*), CT150 (RFM475 *ΔrecQ*), VU118 (RFM475/pPH1243), SB265 (RFM475 *ΔrecA*), VU454 (RFM475 *ΔrecO*) and VU148 (RFM475 *dnaT*). pSK760 carries the *rnhA* gene for RNase HI overproduction. Cells carrying pPH1243 where grown in the presence of IPTG to overproduce topo III.
(PPTX)

Figure S2 Chromosome segregation defects in a *ΔtopA gyrB*(Ts) strain at 24°C. Superimposed images of DIC and fluorescence pictures of DAPI-stained cells grown at 24°C, unless otherwise indicated, as described in Materials and Methods. Size bars are 5 µm. The strains used are all derivatives of RFM475 (*gyrB*(Ts) *ΔtopA*). They are: VU287 (RFM475/pSK760), VU155 (RFM475 *oriC*), CT150 (RFM475 *ΔrecQ*), VU118 (RFM475/pPH1243) and SB265 (RFM475 *ΔrecA*). pSK760 carries the *rnhA* gene for RNase HI overproduction. Cells carrying pPH1243 where grown in the presence of IPTG to overproduce topo III. The length of approximately 150 cells was measured for each strain and the proportion of filaments (arbitrarily cells longer than 7 microns) were determined: RFM475, 76%; RFM475/pSK760, 94%; RFM475 *oriC*, 38%; RFM475 *ΔrecQ*, 32%; RFM475/pPH1243, 28%; RFM475 *ΔrecA*, 29%.
(PPTX)

Figure S3 Topo IV is not overproduced following the deletion of *recQ* in strain RFM475. Cells were grown overnight on LB plates at 37°C. Aliquots were recovered for Western blotting using anti-ParC and anti-ParE antibodies as described by Usongo *et al.* (2013). Strains used are RFM475 *(gyrB*(Ts) *ΔtopA*) and CT150 (RFM475 *ΔrecQ*). The result shown here is representative of three independent experiments. (Usongo V, Tanguay C, Nolent F, Bessong JE, Drolet M (2013) Interplay between type 1A topoisomerases and gyrase in chromosome segregation in *Escherichia coli*. J Bacteriol 195: 1758–1768.).
(PPTX)

Figure S4 The *oriC*::Tn*5* allele can be introduced within *topA* null but not *topA* ^+^ strains. Strains were grown in LB medium to OD_600_ of 0.6 at 37°C. Genomic DNA was prepared essentially as described by Nordman *et al.* (2007). Following genomic DNA extraction, samples were digested with XmnI and electrophoresis was performed in 0.8% agarose in 0.5× TBE at 45V for 24 h at room temperature. After electrophoresis, samples were transferred onto a nitrocellulose membrane (Hybond-N GE Healthcare) and hybridized with a ^32^P-dCTP-labelled probe obtained by PCR using the primers forward 5′- CATTGGCGGGGGTCATGC-3′ and reverse 5′-CTTGCTCTCCAGCGTCGG-3′ corresponding to the *gidA* gene. The bands were visualised with a Phosphorimager Typhoon 9400 (GE Healthcare). The strains used are: RFM443 (wild-type), RFM443 *kan^r^* (wild-type *kan^r^*: a false-positive), RFM475 (*gyrB*(Ts) *ΔtopA*) and VU155 (RFM475 *oriC15::aph*). (Nordman J, Skovgaard, O, Wright (2007) A novel class of mutations that affect DNA replication in *E. coli*. Mol Microbiol 64: 125–138.).
(PPTX)

Figure S5 The *oriC15::aph* mutation complements the growth defect of a *dnaAcos* mutant at 30°C. The LB plates were incubated for the indicated time and at 30, 36 or 42°C as shown. The strains used were: KA441 (*dnaAcos*) and VU194 (KA441 *oriC15::aph*).
(PPTX)

Figure S6 No effects of *recA* or *recQ* deletions on *gyrB*(Ts) *ΔtopA ΔtopB* cells at 40°C. (a) Cells were spotted on LB plates and incubated at 40°C. The LB plates were photographed after 24 and 48 h of incubation. The strains used are all derivative of RFM475 (*gyrB*(Ts) *ΔtopA*). They are: VU306 (RFM475 *ΔtopB*/pSK760), VU333 (RFM475 *ΔtopB*/pSK762c), VU363 (RFM475 *ΔtopB ΔrecQ*/pSK760), VU365 (RFM475 *ΔtopB ΔrecQ*/pSK762c), VU375 (RFM475 *ΔtopB ΔrecA*/pSK760) and VU379 (RFM475 *ΔtopB ΔrecA*/pSK762c). pSK760 carries the *rnhA* gene for RNase HI overproduction, whereas pSK762c carries a mutated and inactive *rnhA gene*. CT170 (*gyrB*(Ts) *ΔtopA ΔtopB*), (b), VU243 (*gyrB*(Ts) *ΔtopA ΔtopB ΔrecA*), (c) and VU205 (*gyrB*(Ts) *ΔtopA ΔtopB ΔrecQ*), (d) cells were prepared for microscopy as described (Usongo *et al*., 2013). Shown are superimposed images of phase contrast and fluorescence pictures of DAPI-stained cells grown at 40°C. (Usongo V, Tanguay C, Nolent F, Bessong JE, Drolet M (2008) Interplay between type 1A topoisomerases and gyrase in chromosome segregation in *Escherichia coli*. J Bacteriol 195:1758–1768.).
(PPTX)

Figure S7 The phenotypes of a *gyrB*(Ts) *ΔtopA ΔtopB* strain are more severe at 30 than 37°C. (a) Cells were spotted on LB plates and incubated for 24 h at the indicated temperature. (b) Cells were prepared for microscopy as described (Usongo *et al*., 2013). Shown are superimposed images of phase contrast and fluorescence pictures of DAPI-stained cells grown at 37 or 30°C as indicated. The strains used are all derivative of RFM475 (*gyrB*(Ts) *ΔtopA*). They are: CT170 (RMF475 *ΔtopB*), VU306 (CT170/pSK760) and VU333 (CT170/pSK762c). pSK760 carries the *rnhA* gene for RNase HI overproduction, whereas pSK762c carries a mutated and inactive *rnhA gene*. (Usongo V, Tanguay C, Nolent F, Bessong JE, Drolet M (2013) Interplay between type 1A topoisomerases and gyrase in chromosome segregation in *Escherichia coli*. J Bacteriol 195:1758–1768.).
(PPTX)

Figure S8 *aph* insertion sites for three suppressor mutants used in this study. The *aph* insertion sites for the *dnaT18::aph* (a), *holC2::aph* (b) and *rne59::aph* (c) alleles are shown. For (a) and (b) we show the nucleotide sequence of the regulatory regions for *dnaT* (promoter has been characterized) and *holC* (promoter unknown) where the *aph* cassette was inserted. In (c) we show the functional domains of the RNase E protein. Note that the *aph* cassette is inserted within the protein scaffold region (position 883 for *rne59::aph*) that is used by RNase E to interact with other proteins to form the RNA degradosome (RhlB helicase, Enolase and PNPase) (for details see Mackie (2013)). (Mackie GA (2013) RNase E: at the interface of bacterial RNA processing and decay. Nat Rev Microbiol 11:45–47.).
(PPTX)

Figure S9 Effect of *dnaT18::aph* allele on the chromosome segregation defects of a *topA rnhA gyrB*(Ts) strain. Superimposed images of DIC and fluorescence pictures of DAPI-stained cells grown at 37°C in LB without IPTG. Size bars are 5 µm. The strains used are all derivatives of RFM475 (*gyrB*(Ts) *ΔtopA*). They are: VU129 (RFM475 *rnhA*/pPH1243) and VU148 (VU129 *dnaT*). pPH1243 carries the *topB* gene under the control of an IPTG-inducible promoter.
(PPTX)

Figure S10 The *ori/ter* ratio is similarly reduced in strains lacking either *rnhA* or *topA*. Growth of the strains, genomic DNA extraction and cutting, and Southern blotting were performed as described in the legend to Figure S4. For the “*ori*” probe, the DNA fragment including the *gidA* gene as described in the legend to Figure S4 was used. For the “*ter*” probe, a DNA fragment including the *cedA* gene (obtained from PCR with the following primers: 5′-GTTACGCGTATCAGGGGC-3′ and 5′-GAGCGACGCCACAGGATG-3′) was used. Strains used were: RFM443 (wild-type), RFM445 (*gyrB*(Ts)), RFM475 (*gyrB(*Ts) *ΔtopA*) and PH379 (*rnhA*). The “*ori*” and “*ter*” bands were visualised and the signal quantified by using a Phosphorimager Typhoon 9400 and the ImageQuant software (GE Healthcare). Shown here are the results of two independent experiments.
(PPTX)

Figure S11 Replication initiation asynchrony conferred by the *holC2::aph* mutation. Rifampicin run-out experiments for flow cytometry analysis were performed as described in Materials and Methods. Cells were grown in M9 minimal medium. The strains used are: RFM445 (*gyrB*(Ts)), RFM475 (*gyrB*(Ts) *ΔtopA*) and VU176 (RFM475 *holC2::aph*).
(PPTX)

Table S1 *Escherichia coli* strains and plasmids used in this work. The strains were constructed as described in Material and Methods.
(DOC)
