# Supplementary material for: Roles of Type 1A Topoisomerases in Genome Maintenance in Escherichia coli
Source: PLoS Genet. 2014 Aug 7;10(8):e1004543. doi: 10.1371/journal.pgen.1004543 (PMC4125114; doi:10.1371/journal.pgen.1004543)
Supplement: Figure S1 — Chromosome segregation defects in a ΔtopA gyrB(Ts) strain at 30°C. Superimposed images of DIC and fluorescence pictures of DAPI-stained cells grown at 30°C, unless otherwise indicated, as described in Materials and Methods. Size bars are 5 µm. The strains used are all derivatives of RFM475 (gyrB(Ts) ΔtopA) except RFM445 (gyrB(Ts)). They are: VU287 (RFM475/pSK760), VU155 (RFM475 oriC), CT150 (RFM475 ΔrecQ), VU118 (RFM475/pPH1243), SB265 (RFM475 ΔrecA), VU454 (RFM475 ΔrecO) and VU148 (RFM475 dnaT). pSK760 carries the rnhA gene for RNase HI overproduction. Cells carrying pPH1243 where grown in the presence of IPTG to overproduce topo III. (PPTX) [file pgen.1004543.s001.pptx]

## Slide 1
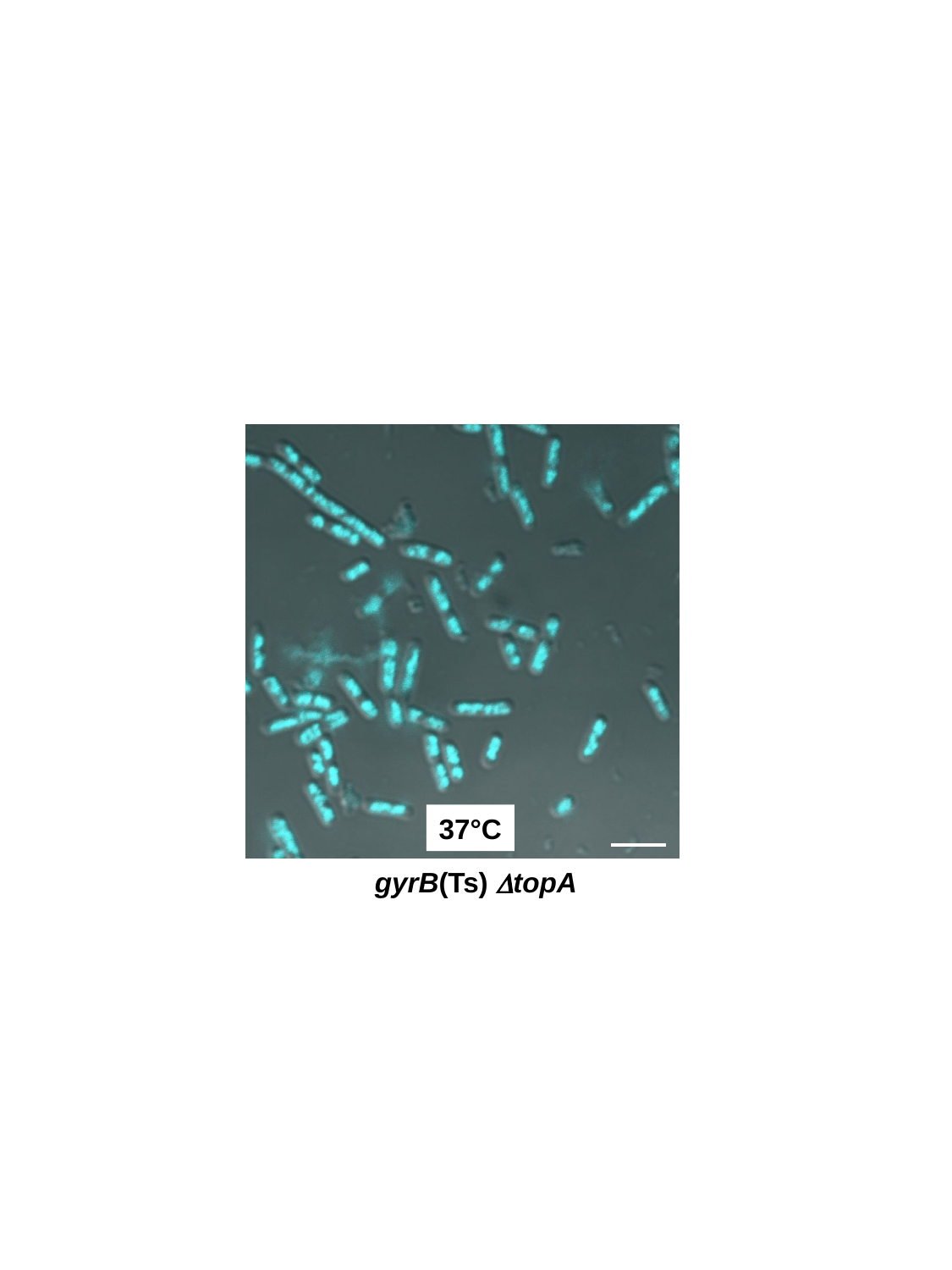

37°C
gyrB(Ts) DtopA

## Slide 2
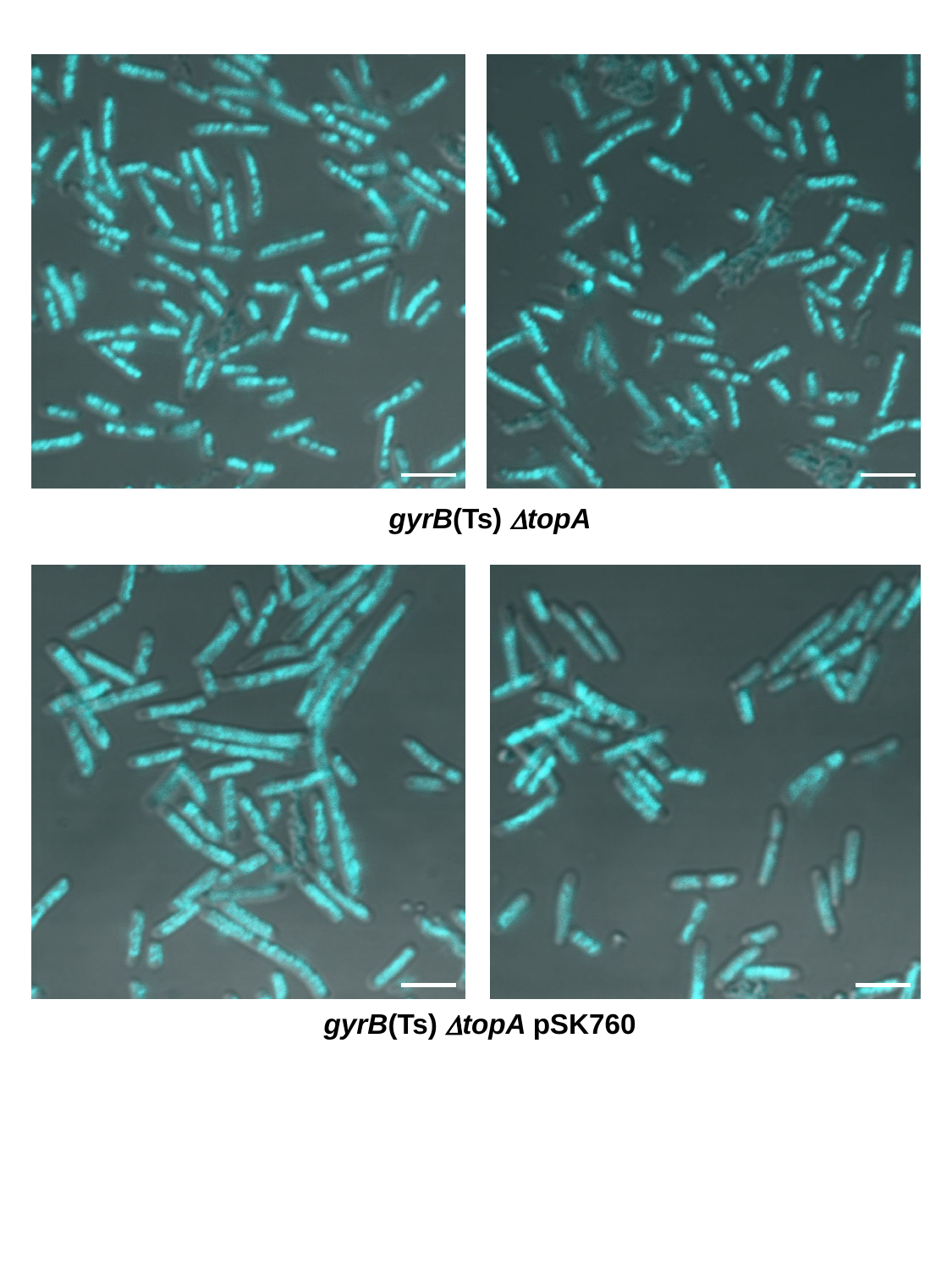

gyrB(Ts) DtopA
gyrB(Ts) DtopA pSK760

## Slide 3
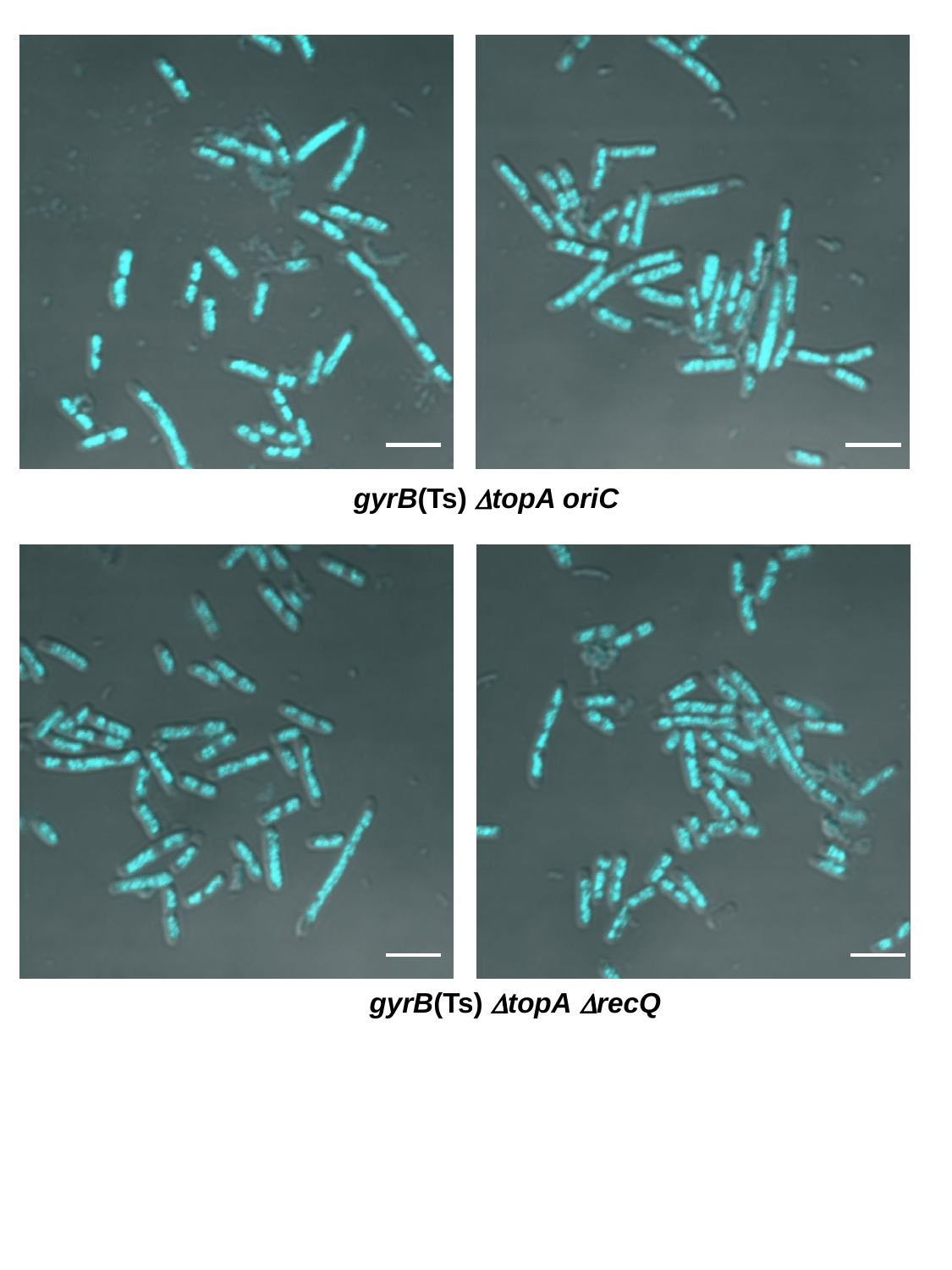

gyrB(Ts) DtopA oriC
gyrB(Ts) DtopA DrecQ

## Slide 4
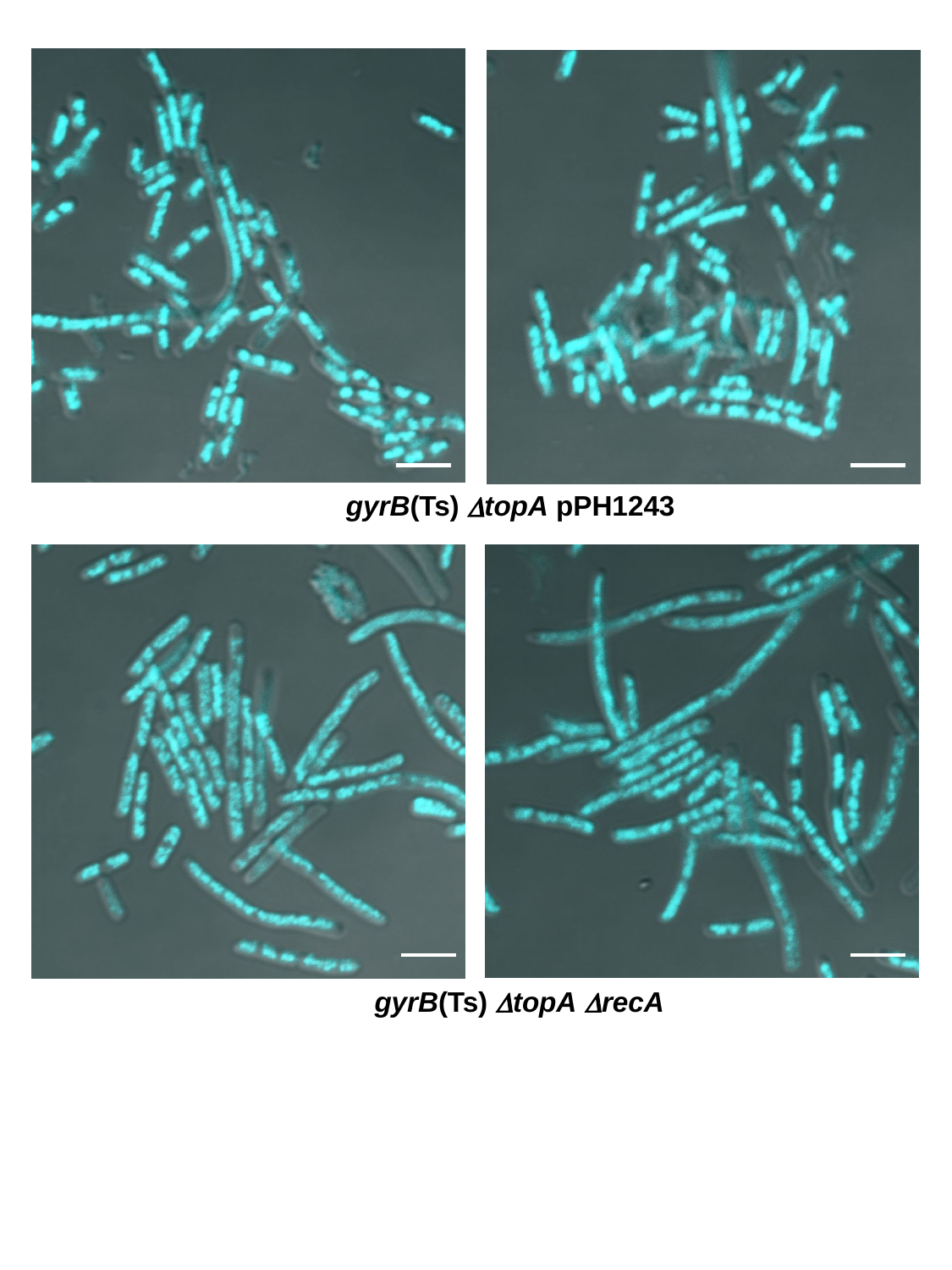

gyrB(Ts) DtopA pPH1243
gyrB(Ts) DtopA DrecA

## Slide 5
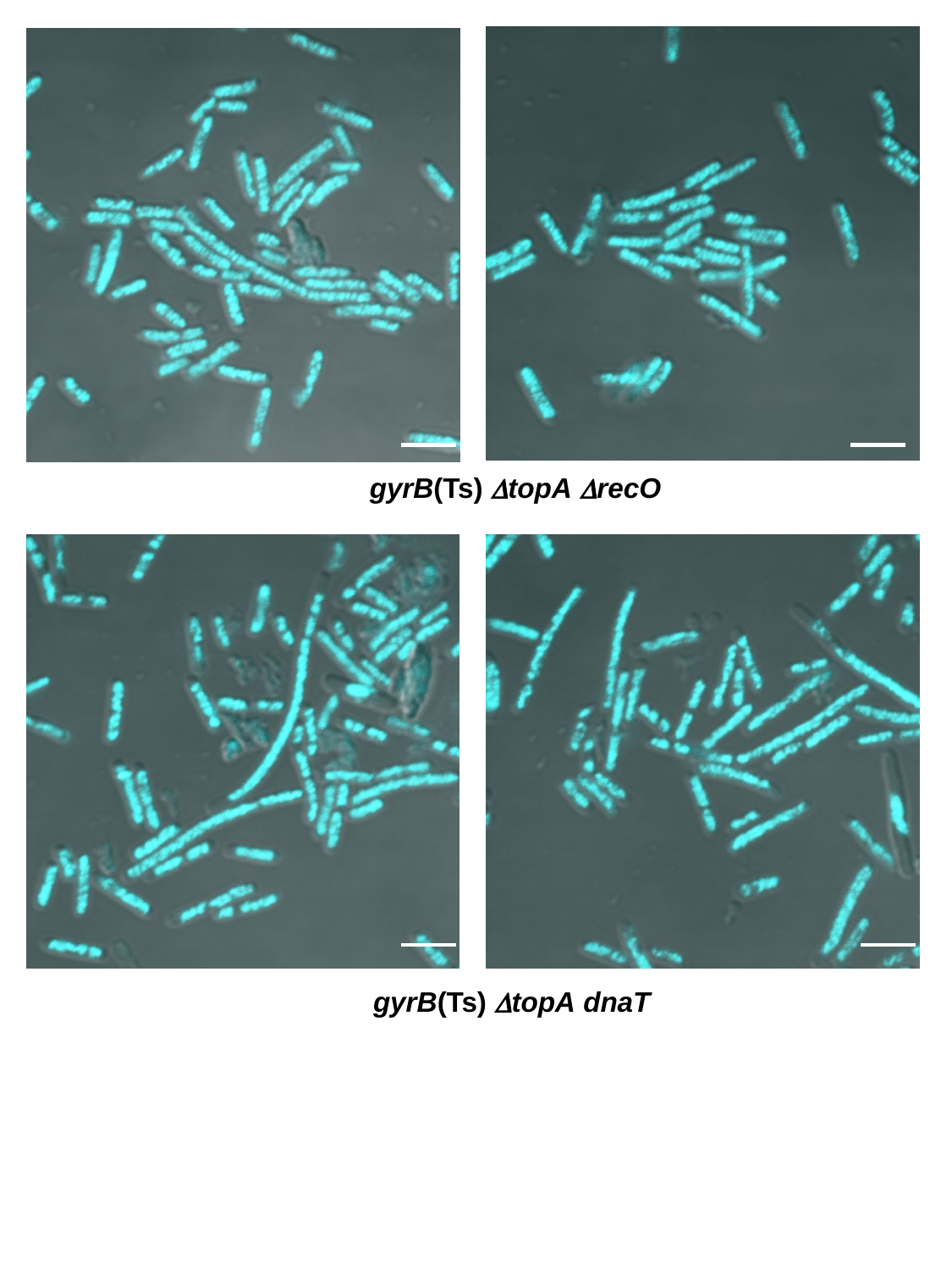

gyrB(Ts) DtopA DrecO
gyrB(Ts) DtopA dnaT
