# Supplementary material for: Roles of Type 1A Topoisomerases in Genome Maintenance in Escherichia coli
Source: PLoS Genet. 2014 Aug 7;10(8):e1004543. doi: 10.1371/journal.pgen.1004543 (PMC4125114; doi:10.1371/journal.pgen.1004543)
Supplement: Figure S2 — Chromosome segregation defects in a ΔtopA gyrB(Ts) strain at 24°C. Superimposed images of DIC and fluorescence pictures of DAPI-stained cells grown at 24°C, unless otherwise indicated, as described in Materials and Methods. Size bars are 5 µm. The strains used are all derivatives of RFM475 (gyrB(Ts) ΔtopA). They are: VU287 (RFM475/pSK760), VU155 (RFM475 oriC), CT150 (RFM475 ΔrecQ), VU118 (RFM475/pPH1243) and SB265 (RFM475 ΔrecA). pSK760 carries the rnhA gene for RNase HI overproduction. Cells carrying pPH1243 where grown in the presence of IPTG to overproduce topo III. The length of approximately 150 cells was measured for each strain and the proportion of filaments (arbitrarily cells longer than 7 microns) were determined: RFM475, 76%; RFM475/pSK760, 94%; RFM475 oriC, 38%; RFM475 ΔrecQ, 32%; RFM475/pPH1243, 28%; RFM475 ΔrecA, 29%. (PPTX) [file pgen.1004543.s002.pptx]

## Slide 1
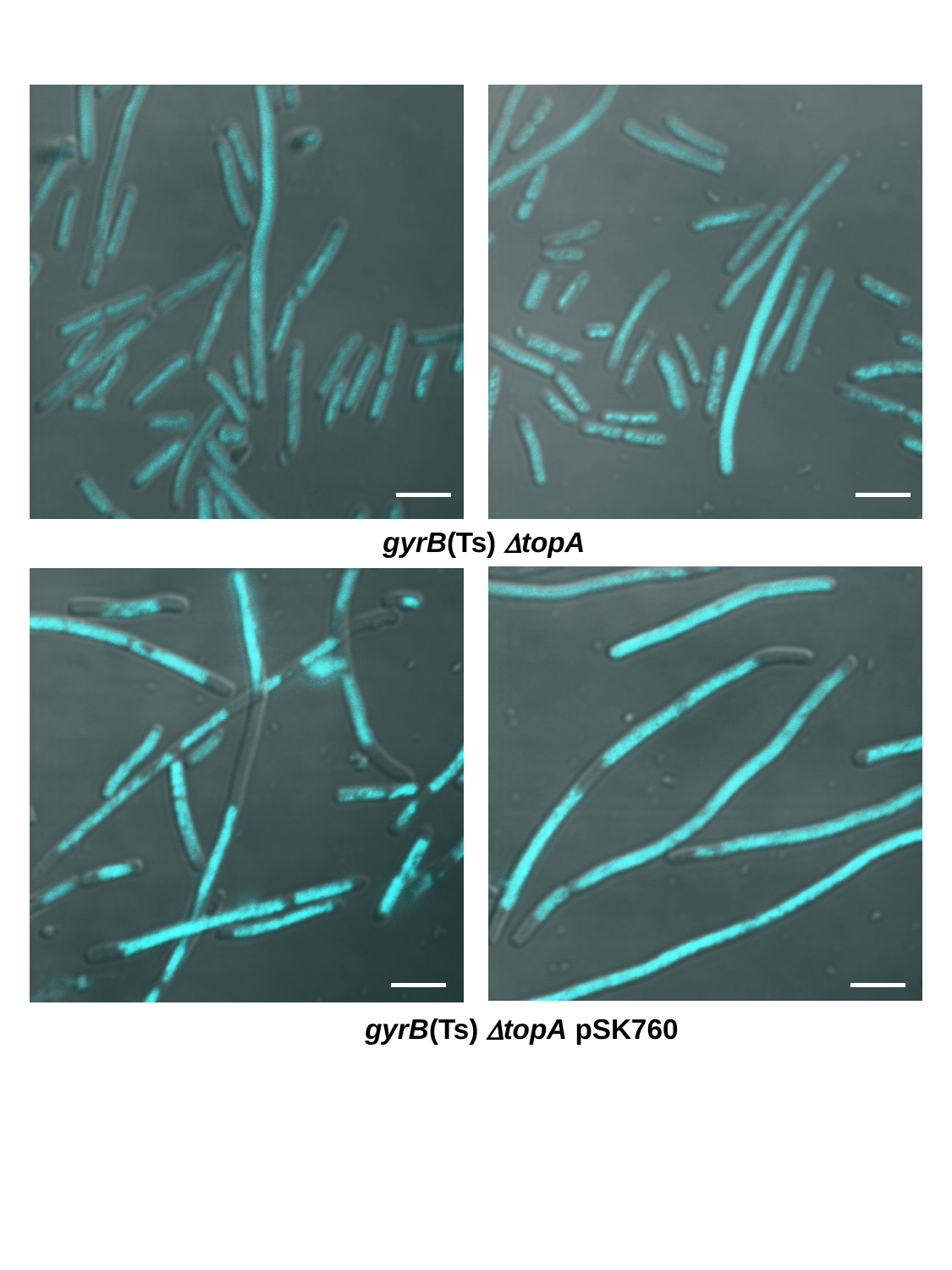

gyrB(Ts) DtopA
gyrB(Ts) DtopA pSK760

## Slide 2
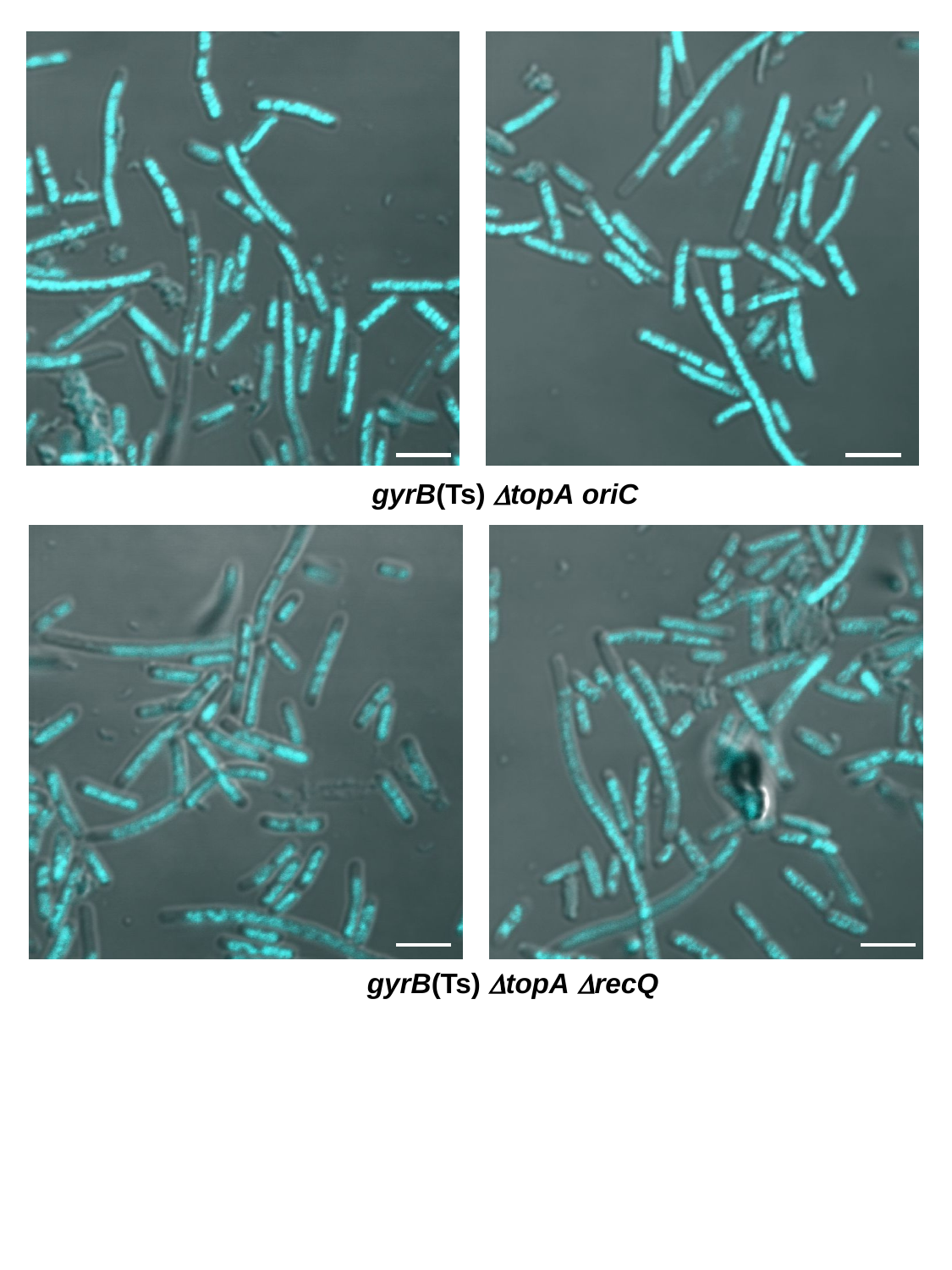

gyrB(Ts) DtopA oriC
gyrB(Ts) DtopA DrecQ

## Slide 3
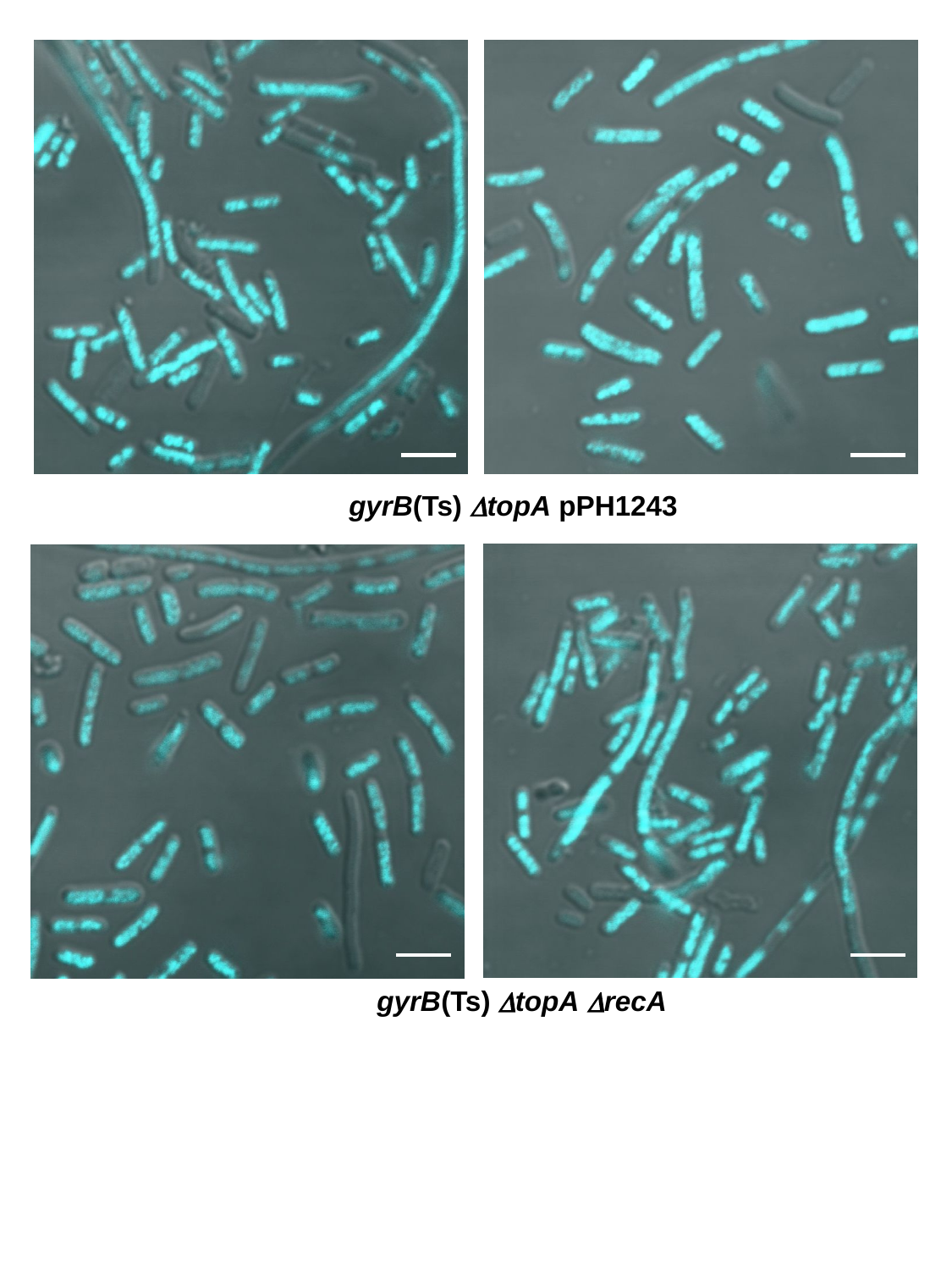

gyrB(Ts) DtopA pPH1243
gyrB(Ts) DtopA DrecA
