# Supplementary figures and images for: Roles of Type 1A Topoisomerases in Genome Maintenance in Escherichia coli
Source: PLoS Genet. 2014 Aug 7;10(8):e1004543. doi: 10.1371/journal.pgen.1004543 (PMC4125114; doi:10.1371/journal.pgen.1004543)

## Slide 1
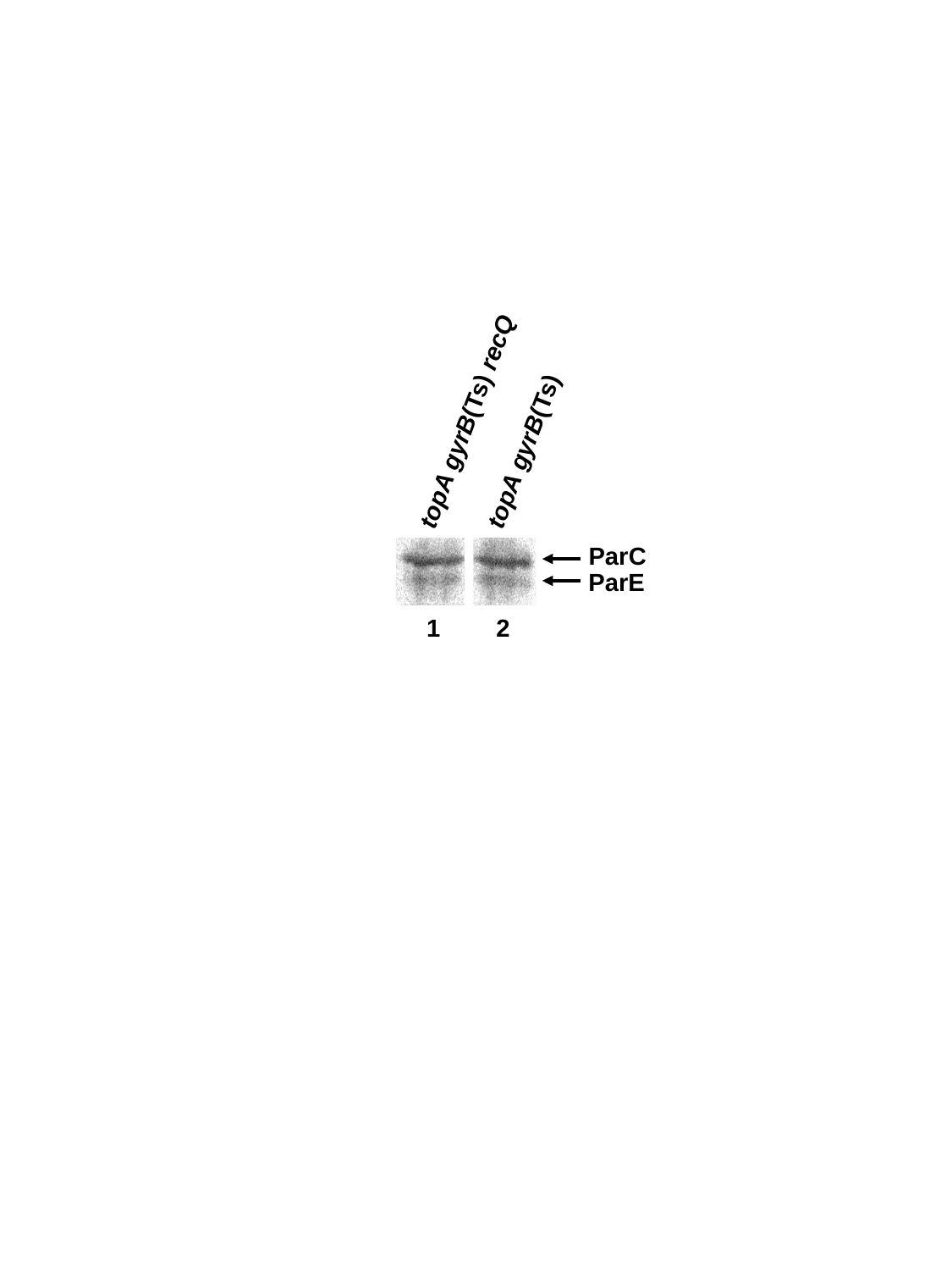

topA gyrB(Ts) recQ
topA gyrB(Ts)
ParC
ParE
1 2

Supplement: Figure S3 — Topo IV is not overproduced following the deletion of recQ in strain RFM475. Cells were grown overnight on LB plates at 37°C. Aliquots were recovered for Western blotting using anti-ParC and anti-ParE antibodies as described by Usongo et al. (2013). Strains used are RFM475 (gyrB(Ts) ΔtopA) and CT150 (RFM475 ΔrecQ). The result shown here is representative of three independent experiments. (Usongo V, Tanguay C, Nolent F, Bessong JE, Drolet M (2013) Interplay between type 1A topoisomerases and gyrase in chromosome segregation in Escherichia coli. J Bacteriol 195: 1758–1768.). (PPTX) [file pgen.1004543.s003.pptx]

## Slide 1
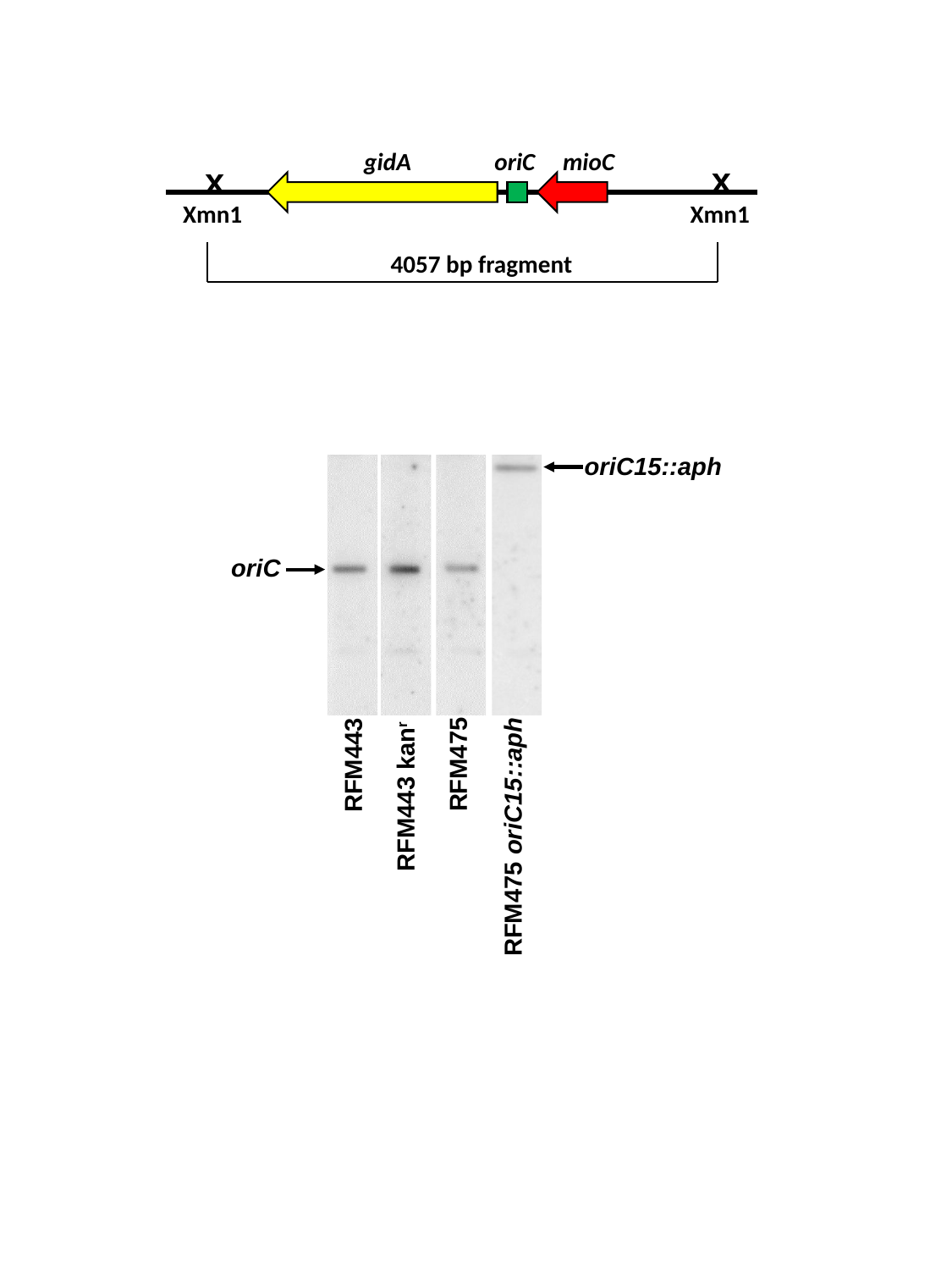

gidA
oriC
mioC
x
x
Xmn1
Xmn1
4057 bp fragment
 oriC15::aph
oriC
RFM443
RFM475
RFM443 kanr
RFM475 oriC15::aph

Supplement: Figure S4 — The oriC::Tn5 allele can be introduced within topA null but not topA + strains. Strains were grown in LB medium to OD600 of 0.6 at 37°C. Genomic DNA was prepared essentially as described by Nordman et al. (2007). Following genomic DNA extraction, samples were digested with XmnI and electrophoresis was performed in 0.8% agarose in 0.5× TBE at 45V for 24 h at room temperature. After electrophoresis, samples were transferred onto a nitrocellulose membrane (Hybond-N GE Healthcare) and hybridized with a 32P-dCTP-labelled probe obtained by PCR using the primers forward 5′- CATTGGCGGGGGTCATGC-3′ and reverse 5′-CTTGCTCTCCAGCGTCGG-3′ corresponding to the gidA gene. The bands were visualised with a Phosphorimager Typhoon 9400 (GE Healthcare). The strains used are: RFM443 (wild-type), RFM443 kanr (wild-type kanr: a false-positive), RFM475 (gyrB(Ts) ΔtopA) and VU155 (RFM475 oriC15::aph). (Nordman J, Skovgaard, O, Wright (2007) A novel class of mutations that affect DNA replication in E. coli. Mol Microbiol 64: 125–138.). (PPTX) [file pgen.1004543.s004.pptx]

## Slide 1
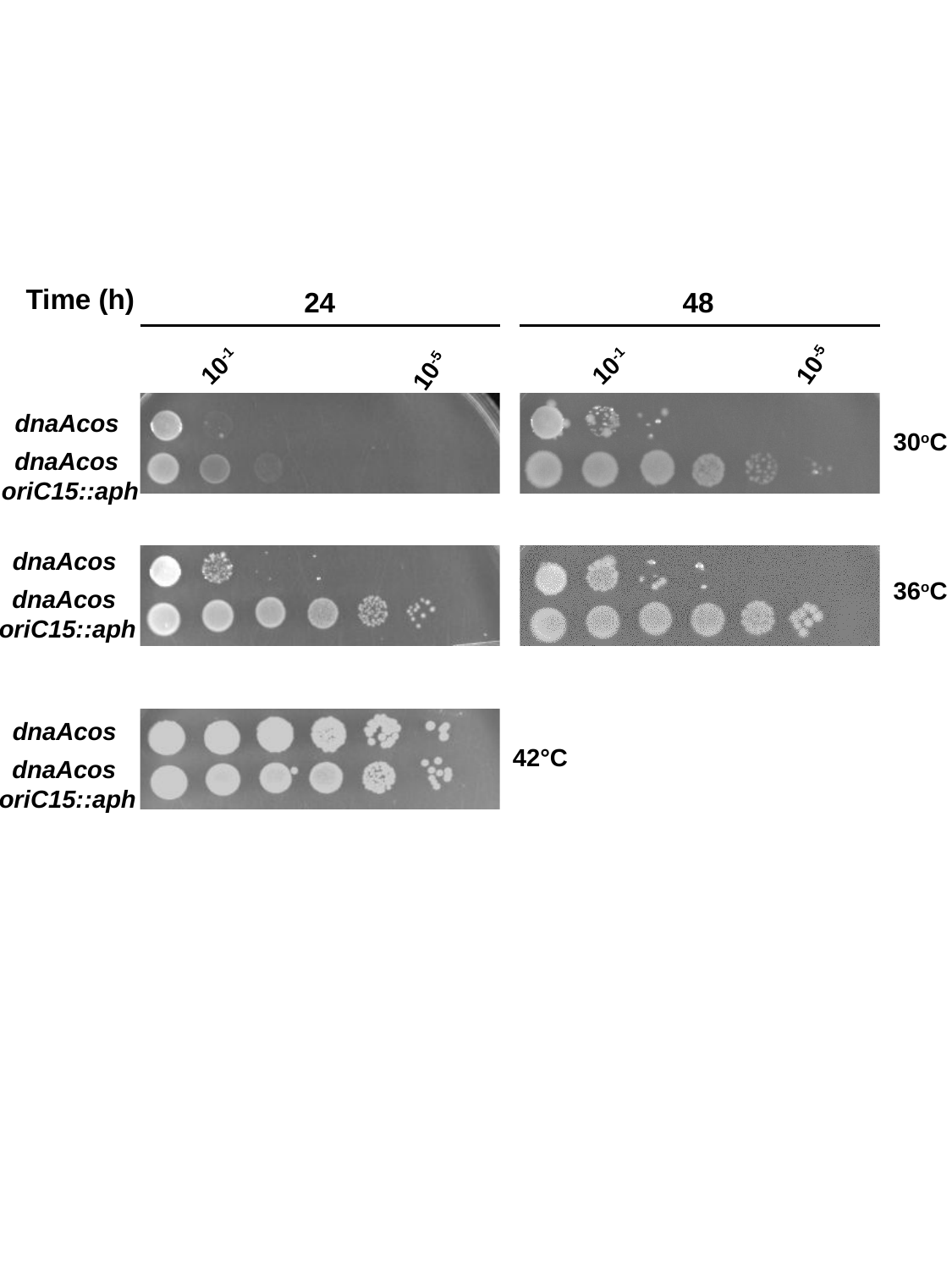

Time (h)
24
48
 10-1
 10-1
10-5
10-5
dnaAcos
30oC
dnaAcos
oriC15::aph
dnaAcos
36oC
dnaAcos
oriC15::aph
dnaAcos
42°C
dnaAcos
oriC15::aph

Supplement: Figure S5 — The oriC15::aph mutation complements the growth defect of a dnaAcos mutant at 30°C. The LB plates were incubated for the indicated time and at 30, 36 or 42°C as shown. The strains used were: KA441 (dnaAcos) and VU194 (KA441 oriC15::aph). (PPTX) [file pgen.1004543.s005.pptx]

## Slide 1
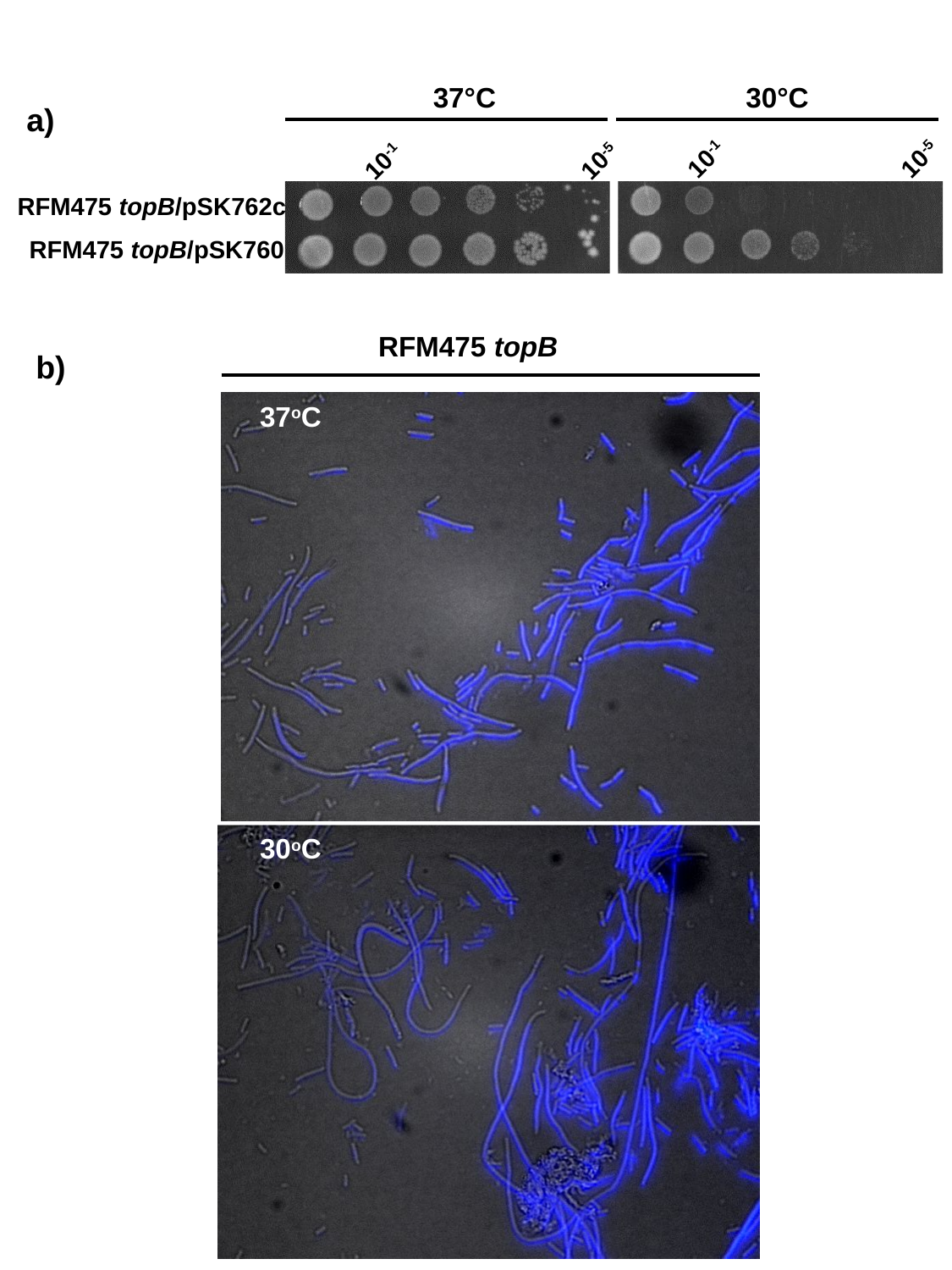

37°C
30°C
a)
 10-1
 10-5
 10-5
 10-1
RFM475 topB/pSK762c
RFM475 topB/pSK760
RFM475 topB
b)
37oC
30oC

Supplement: Figure S7 — The phenotypes of a gyrB(Ts) ΔtopA ΔtopB strain are more severe at 30 than 37°C. (a) Cells were spotted on LB plates and incubated for 24 h at the indicated temperature. (b) Cells were prepared for microscopy as described (Usongo et al., 2013). Shown are superimposed images of phase contrast and fluorescence pictures of DAPI-stained cells grown at 37 or 30°C as indicated. The strains used are all derivative of RFM475 (gyrB(Ts) ΔtopA). They are: CT170 (RMF475 ΔtopB), VU306 (CT170/pSK760) and VU333 (CT170/pSK762c). pSK760 carries the rnhA gene for RNase HI overproduction, whereas pSK762c carries a mutated and inactive rnhA gene. (Usongo V, Tanguay C, Nolent F, Bessong JE, Drolet M (2013) Interplay between type 1A topoisomerases and gyrase in chromosome segregation in Escherichia coli. J Bacteriol 195:1758–1768.). (PPTX) [file pgen.1004543.s007.pptx]

## Slide 1
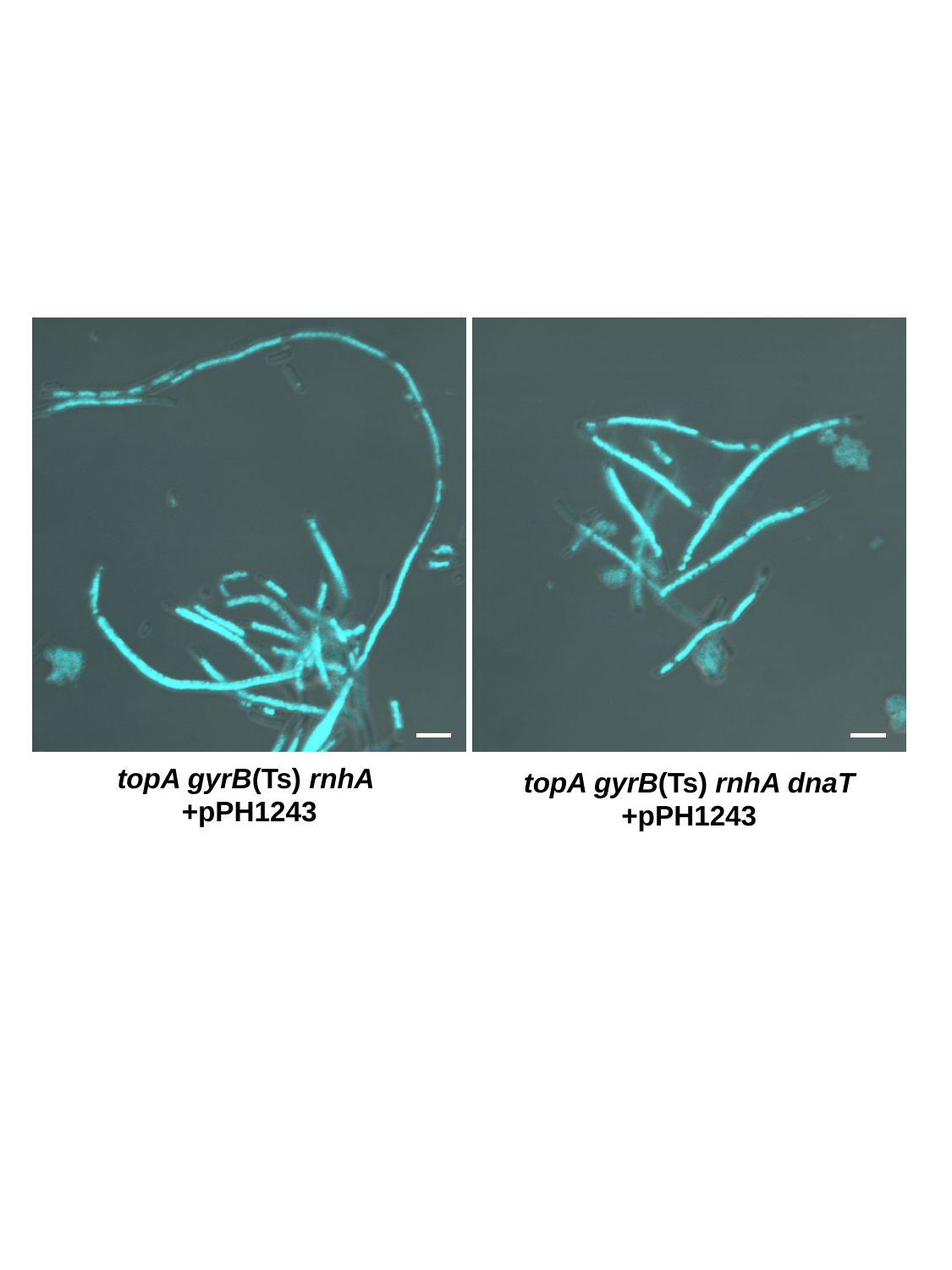

topA gyrB(Ts) rnhA
+pPH1243
topA gyrB(Ts) rnhA dnaT
+pPH1243

Supplement: Figure S9 — Effect of dnaT18::aph allele on the chromosome segregation defects of a topA rnhA gyrB(Ts) strain. Superimposed images of DIC and fluorescence pictures of DAPI-stained cells grown at 37°C in LB without IPTG. Size bars are 5 µm. The strains used are all derivatives of RFM475 (gyrB(Ts) ΔtopA). They are: VU129 (RFM475 rnhA/pPH1243) and VU148 (VU129 dnaT). pPH1243 carries the topB gene under the control of an IPTG-inducible promoter. (PPTX) [file pgen.1004543.s009.pptx]

## Slide 1
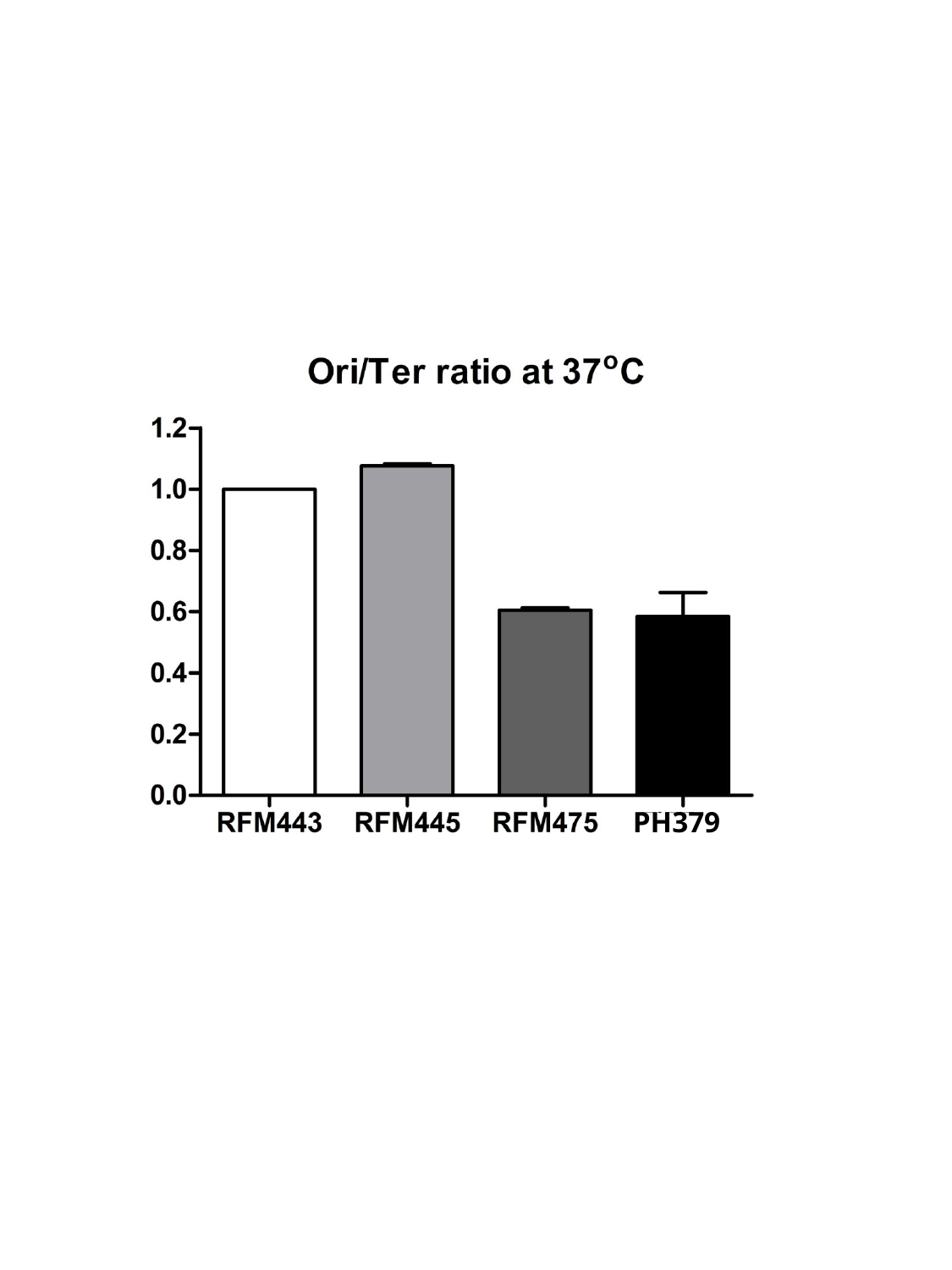

PH379

Supplement: Figure S10 — The ori/ter ratio is similarly reduced in strains lacking either rnhA or topA. Growth of the strains, genomic DNA extraction and cutting, and Southern blotting were performed as described in the legend to Figure S4. For the “ori” probe, the DNA fragment including the gidA gene as described in the legend to Figure S4 was used. For the “ter” probe, a DNA fragment including the cedA gene (obtained from PCR with the following primers: 5′-GTTACGCGTATCAGGGGC-3′ and 5′-GAGCGACGCCACAGGATG-3′) was used. Strains used were: RFM443 (wild-type), RFM445 (gyrB(Ts)), RFM475 (gyrB(Ts) ΔtopA) and PH379 (rnhA). The “ori” and “ter” bands were visualised and the signal quantified by using a Phosphorimager Typhoon 9400 and the ImageQuant software (GE Healthcare). Shown here are the results of two independent experiments. (PPTX) [file pgen.1004543.s010.pptx]

## Slide 1
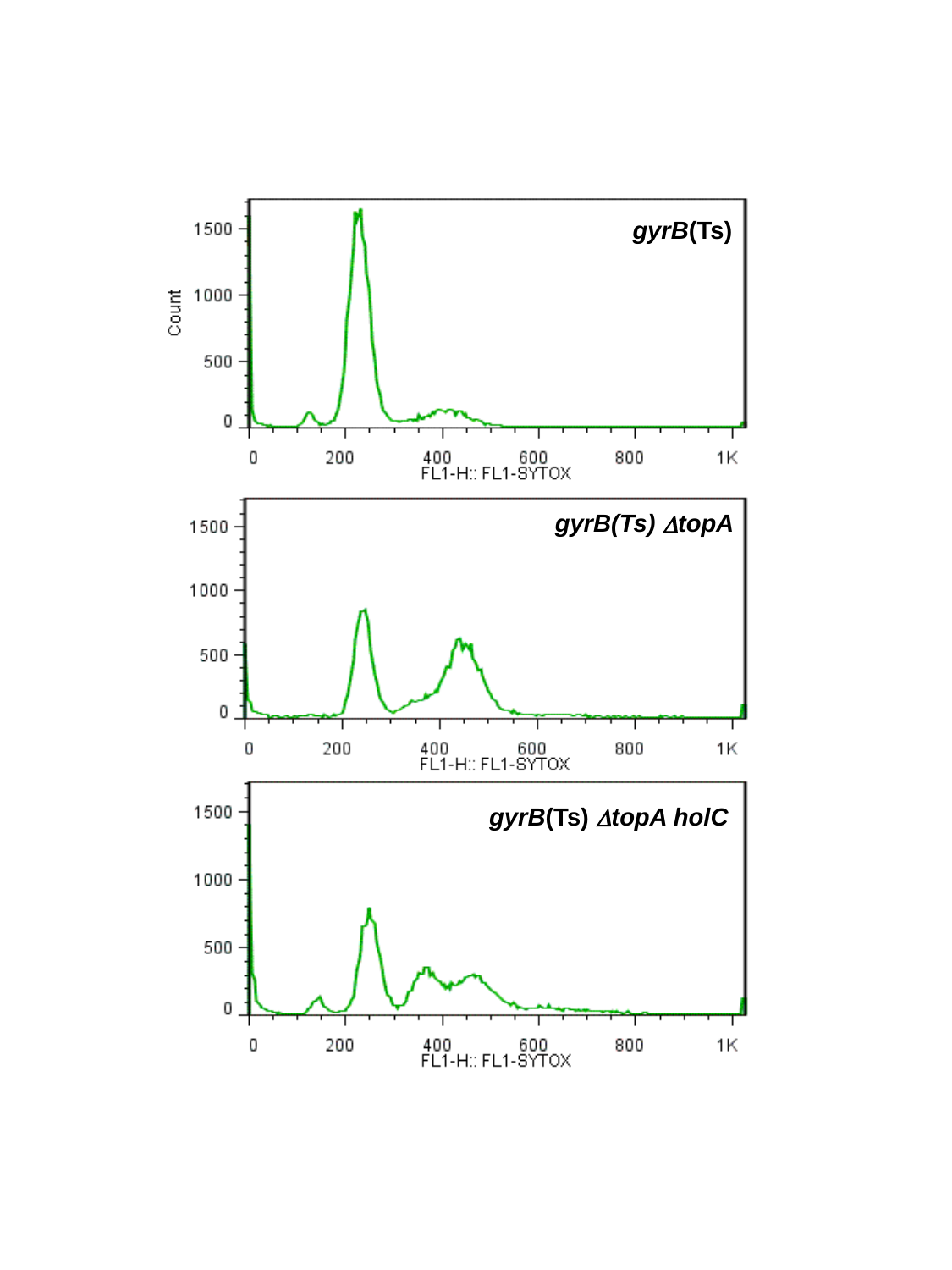

gyrB(Ts)
gyrB(Ts) DtopA
gyrB(Ts) DtopA holC

Supplement: Figure S11 — Replication initiation asynchrony conferred by the holC2::aph mutation. Rifampicin run-out experiments for flow cytometry analysis were performed as described in Materials and Methods. Cells were grown in M9 minimal medium. The strains used are: RFM445 (gyrB(Ts)), RFM475 (gyrB(Ts) ΔtopA) and VU176 (RFM475 holC2::aph). (PPTX) [file pgen.1004543.s011.pptx]
