# Supplementary material for: Roles of Type 1A Topoisomerases in Genome Maintenance in Escherichia coli
Source: PLoS Genet. 2014 Aug 7;10(8):e1004543. doi: 10.1371/journal.pgen.1004543 (PMC4125114; doi:10.1371/journal.pgen.1004543)
Supplement: Figure S6 — No effects of recA or recQ deletions on gyrB(Ts) ΔtopA ΔtopB cells at 40°C. (a) Cells were spotted on LB plates and incubated at 40°C. The LB plates were photographed after 24 and 48 h of incubation. The strains used are all derivative of RFM475 (gyrB(Ts) ΔtopA). They are: VU306 (RFM475 ΔtopB/pSK760), VU333 (RFM475 ΔtopB/pSK762c), VU363 (RFM475 ΔtopB ΔrecQ/pSK760), VU365 (RFM475 ΔtopB ΔrecQ/pSK762c), VU375 (RFM475 ΔtopB ΔrecA/pSK760) and VU379 (RFM475 ΔtopB ΔrecA/pSK762c). pSK760 carries the rnhA gene for RNase HI overproduction, whereas pSK762c carries a mutated and inactive rnhA gene. CT170 (gyrB(Ts) ΔtopA ΔtopB), (b), VU243 (gyrB(Ts) ΔtopA ΔtopB ΔrecA), (c) and VU205 (gyrB(Ts) ΔtopA ΔtopB ΔrecQ), (d) cells were prepared for microscopy as described (Usongo et al., 2013). Shown are superimposed images of phase contrast and fluorescence pictures of DAPI-stained cells grown at 40°C. (Usongo V, Tanguay C, Nolent F, Bessong JE, Drolet M (2008) Interplay between type 1A topoisomerases and gyrase in chromosome segregation in Escherichia coli. J Bacteriol 195:1758–1768.). (PPTX) [file pgen.1004543.s006.pptx]

## Slide 1
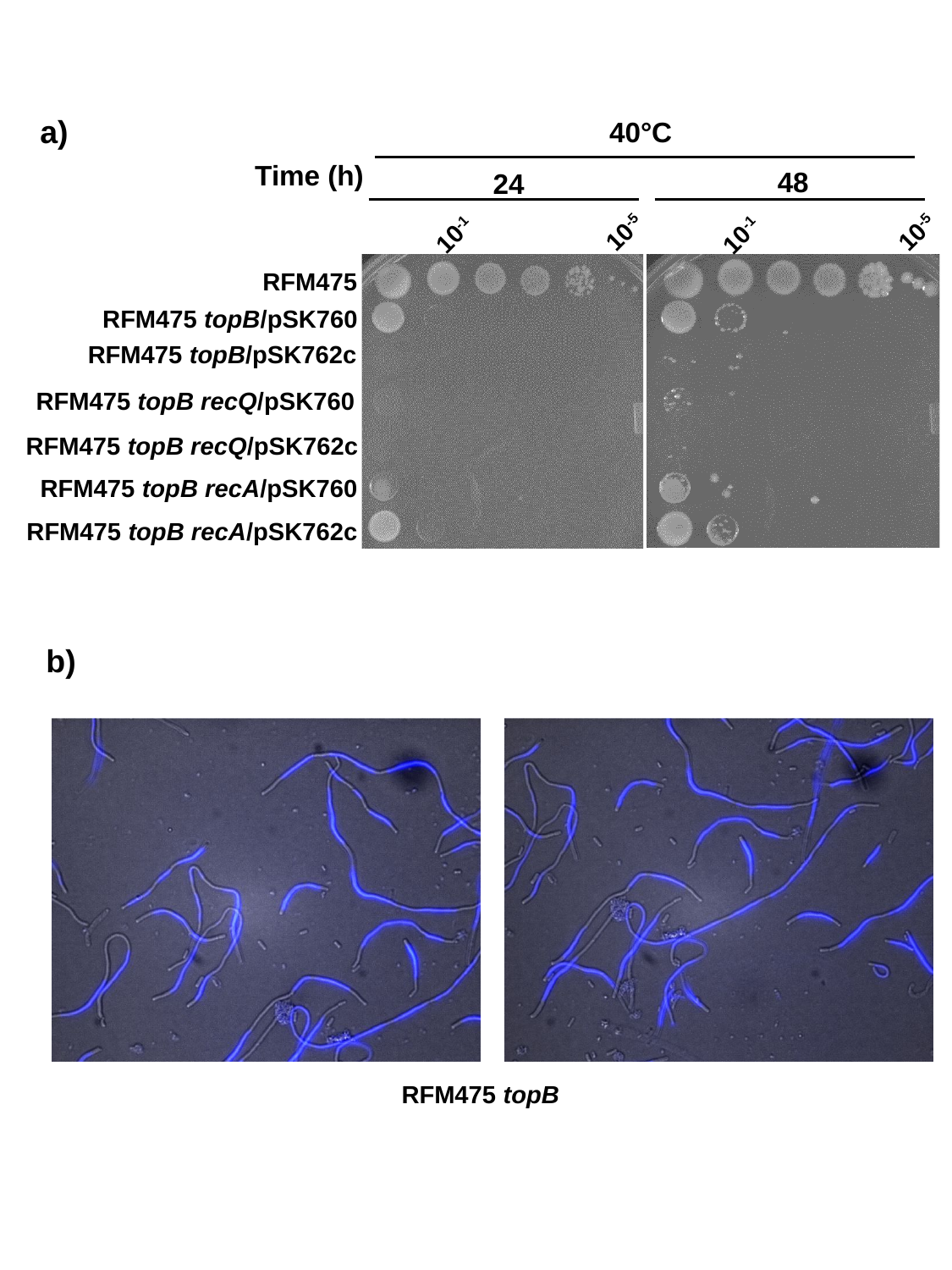

a)
40°C
Time (h)
24
48
 10-1
10-5
10-5
 10-1
RFM475
RFM475 topB/pSK760
RFM475 topB/pSK762c
10-5
RFM475 topB recQ/pSK760
RFM475 topB recQ/pSK762c
RFM475 topB recA/pSK760
RFM475 topB recA/pSK762c
b)
RFM475 topB

## Slide 2
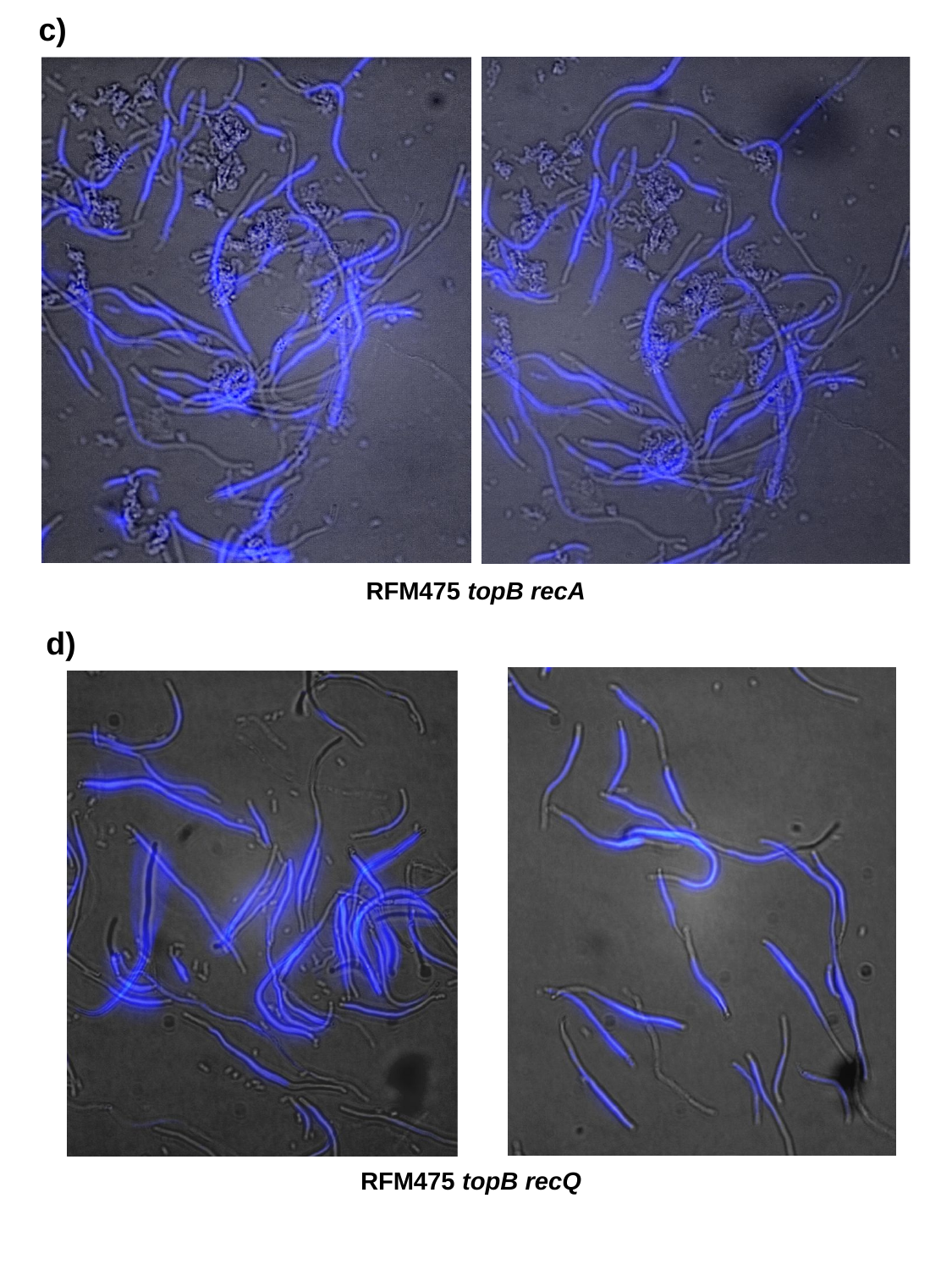

c)
RFM475 topB recA
d)
RFM475 topB recQ
