# Supplementary material for: Roles of Type 1A Topoisomerases in Genome Maintenance in Escherichia coli
Source: PLoS Genet. 2014 Aug 7;10(8):e1004543. doi: 10.1371/journal.pgen.1004543 (PMC4125114; doi:10.1371/journal.pgen.1004543)
Supplement: Figure S8 — aph insertion sites for three suppressor mutants used in this study. The aph insertion sites for the dnaT18::aph (a), holC2::aph (b) and rne59::aph (c) alleles are shown. For (a) and (b) we show the nucleotide sequence of the regulatory regions for dnaT (promoter has been characterized) and holC (promoter unknown) where the aph cassette was inserted. In (c) we show the functional domains of the RNase E protein. Note that the aph cassette is inserted within the protein scaffold region (position 883 for rne59::aph) that is used by RNase E to interact with other proteins to form the RNA degradosome (RhlB helicase, Enolase and PNPase) (for details see Mackie (2013)). (Mackie GA (2013) RNase E: at the interface of bacterial RNA processing and decay. Nat Rev Microbiol 11:45–47.). (PPTX) [file pgen.1004543.s008.pptx]

## Slide 1
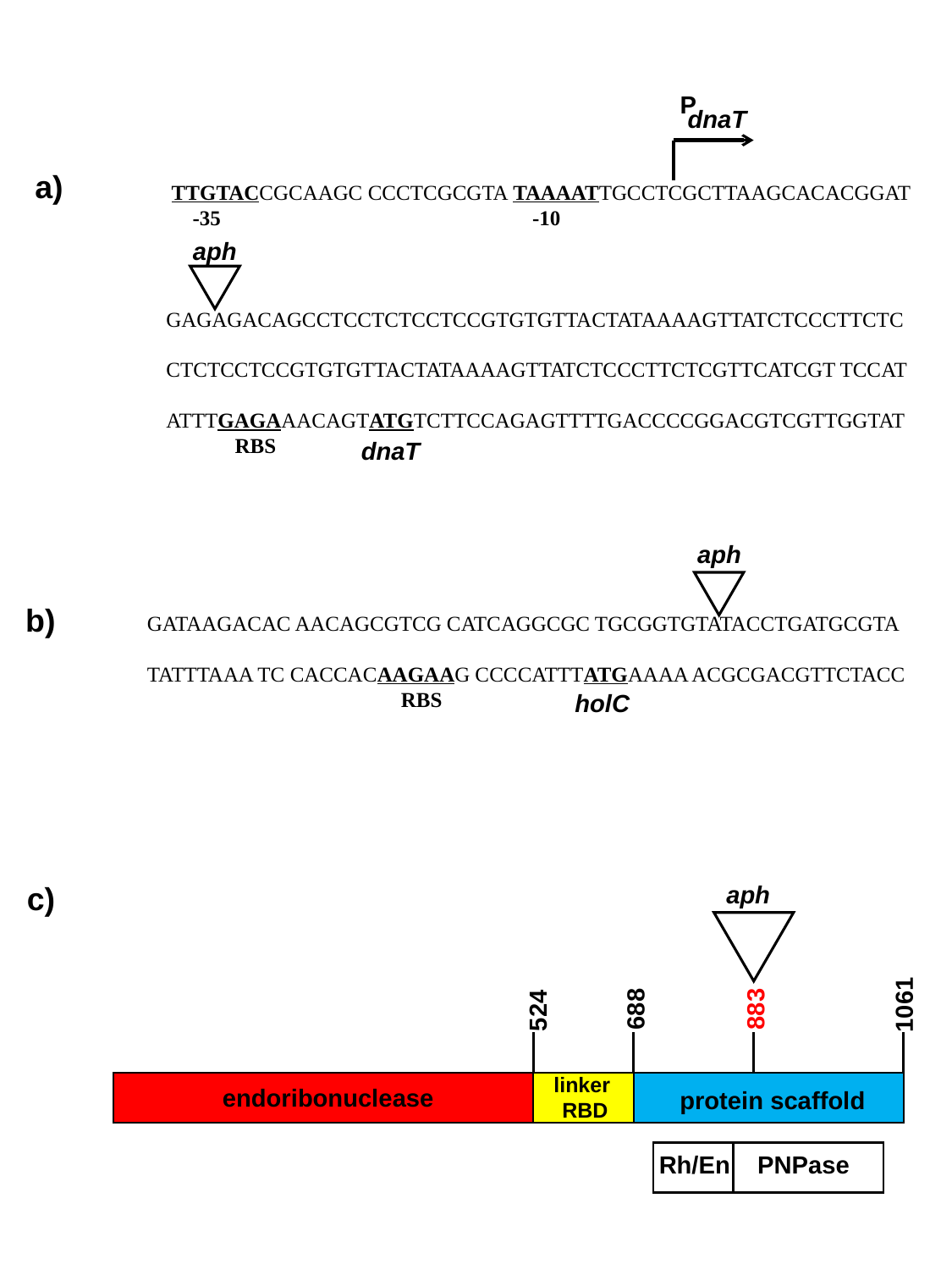

P
dnaT
a)
 TTGTACCGCAAGC CCCTCGCGTA TAAAATTGCCTCGCTTAAGCACACGGAT
 -35 -10
GAGAGACAGCCTCCTCTCCTCCGTGTGTTACTATAAAAGTTATCTCCCTTCTC
CTCTCCTCCGTGTGTTACTATAAAAGTTATCTCCCTTCTCGTTCATCGT TCCAT
ATTTGAGAAACAGTATGTCTTCCAGAGTTTTGACCCCGGACGTCGTTGGTAT
 RBS
aph
dnaT
GATAAGACAC AACAGCGTCG CATCAGGCGC TGCGGTGTATACCTGATGCGTA
TATTTAAA TC CACCACAAGAAG CCCCATTTATGAAAA ACGCGACGTTCTACC
		RBS
aph
b)
holC
c)
aph
1061
688
883
524
linker
RBD
endoribonuclease
protein scaffold
Rh/En
PNPase
