# Supplementary material for: Roles of Type 1A Topoisomerases in Genome Maintenance in Escherichia coli
Source: PLoS Genet. 2014 Aug 7;10(8):e1004543. doi: 10.1371/journal.pgen.1004543 (PMC4125114; doi:10.1371/journal.pgen.1004543)
Supplement: Table S1 — Escherichia coli strains and plasmids used in this work. The strains were constructed as described in Material and Methods. (DOC) [file pgen.1004543.s012.doc]

**Table S1** *E. coli* strains and plasmids used.

| Name | Genotype or Relevant Genotype | Reference or Source |
| --- | --- | --- |
| MA251  CL581  CT150  CT170  JE35 | *gyrB221* (Cou^r^) *gyrB203*(Ts)  *zie-3163*::Tn*10kan topA20*::Tn*10*  *thyA36*, *deoC2*, *IN(rrnD-rrnE)I, rph?*, *recQ6215* (sub cat 883 for cdn 19-606) *recQ6215::cam*  RFM475 *recQ6215::cam*  RFM475 *ΔtopB::kan*  PH379 *dnaA46*(Ts) *tnaA*::Tn*10* | [1]  Justin Courcelle  RFM475 x P1(CL581)  [2]  PH379 x P1(MD48) |
| JE36  JE119  JW0461-2  JW1752-1 | JE35 *rne59*::*aph*  MD48 *rne59*::*aph*  *ΔrecR776::kan*  *ΔtopB761::kan* | This work^a^  This work^a^  [3]  [3] |
| JW2549-1 | *ΔrecO737::kan* | [3] |
| JW2860-1  KA441  MD48 | *ΔrecJ743::kan*  *ilv thyA thr tyrA*(Am) *trpE9829*(Am)  *metE deo supF6*(Ts) *dnaAcos tna***::**Tn*10*  MC4100 *dnaA46*(Ts) *tnaA*::Tn*10* | [3]  [4]  Lab collection |
| PH379  RFM443 | *rnhA ::cam*  *rpsL galK2 ∆lac74* | [5]  [6] |
| RFM445  RFM475  SB262  SB264  SB265  SB362  VU29  VU118  VU129  VU148  VU155  VU176  VU188  VU194  VU200  VU204  VU205  VU243  VU287  VU299  VU306  VU333  VU349  VU363  VU365  VU375  VU379  VU403  VU409  VU414  VU416  VU421  VU422  VU425  VU441  VU454  VU458  VU464  VU468  VU469  VU473  VU479  VU492  VU501  pPH1243  pSK760  pSK762c | *rpsL galK2 gyrB221* (Cou^r^) *gyrB203*(Ts) *Δlac74*  *rpsL galK2 gyrB221* (Cou^r^) *gyrB203*(Ts) *Δlac74 ∆(topA cysB)204*  RFM475 *recB268*::Tn*10*  RFM445 *ΔrecA306 srlR301*::Tn*10*  RFM475 *ΔrecA306 srlR301*::Tn*10*  RFM475 *lexA3* *malF*::Tn10  RFM443 *ΔrecA306 srlR301*::Tn*10*  RFM475 pPH1243  VU118 *rnhA::cam*  VU129 *dnaT18::aph*  RFM475 *oriC15::aph*  RFM475 *holC2::aph*  RFM475 *dnaT18::aph*  KA441 *oriC15::aph*  JE35 *dnaT18::aph*  MD48 *dnaT18::aph*  CT150 *ΔtopB::kan*  CT170 *ΔrecA306 srlR301*::Tn*10*  RFM475 pSK760  RFM475 pSK762c  CT170 pSK760  CT170 pSK762c  RFM475 *ΔrecJ743::kan*  VU205 pSK760  VU205 pSK762c  VU243 pSK760  VU243 pSK762c  RFM445 *ΔtopB761::kan*  RFM445 *ΔtopB*  VU409  *dnaT18::aph*  VU409  *rne59*::*aph*  VU409 *topA20*::Tn*10*  VU421 pSK760  VU421 pSK762c  VU414 *topA20*::Tn*10*  RFM475 *ΔrecO737::kan*  RFM475 *ΔrecR776::kan*  CT150 pPH1243  VU409 *holC2::aph*  VU468 *topA20*::Tn*10*  VU416 *topA20*::Tn*10*  SB265 pPH1243  CT150 *ΔrecA306 srlR301*::Tn*10*  CT150  *lexA3* *malF*::Tn10  *topB* under the control of  IPTG-inducible promoter  *rnhA* gene with its own promoter  like pSK760 but *rnhA* is mutated and  inactive | [6]  [6]  This work  Lab collection  RFM475 x P1(VU29)  This work  Lab collection  This work  VU118 x P1(PH379)  This work^a^  This work^a^  This work^a^  This work^a^  This work^a^  This work^a^  This work^a^  CT150 x P1(DM4100 *ΔtopB::kan)*  CT170 x P1(VU29)  This work  This work  This work  This work  RFM475 x P1(JW2860-1)  This work  This work  This work  This work  RFM445 x P1( JW1752-1)  VU403, *kan* removed by pCP20 [7]  This work^a^  This work^a^  VU409 x P1(MA251)  This work  This work  VU414 x P1(MA251)  RFM475 x P1(JW2549-1)  RFM475 x P1(JW0461-2)  This work  This work^a^  VU468 x P1(MA251)  VU416 x P1(MA251)  This work  This work  This work  [8]  [6]  [6] |

^a^ The P1*vir* phage lysates used to transfer the various insertion mutants described in this study were obtained from the original suppressed clones obtained at 40^o^C (see Material and Methods)

**Supplemental References**

1. Hraiky C, Raymond MA, Drolet M (2000) RNase H overproduction corrects a defect at the level of transcription elongation during rRNA synthesis in the absence of DNA topoisomerase I in *Escherichia coli.* J Biol Chem 275:11257-11263.
2. Usongo V, Tanguay C, Nolent F, Egbe Bessong J, Drolet M (2013) Interplay between type 1A topoisomerases and gyrase in chromosome segregation in *Escherichia coli*. J Bacteriol 195:1758-1768.
3. Baba T, Ara T, Hasegawa M, Takai Y, Okumura Y, et al. (2006) Construction of *Escherichia coli* K-12 in-frame, single-gene knockout mutants: the Keio collection. Mol Syst Biol 2: 2006.0008.
4. Katayama T, Kornberg A (1994) Hyperactive initiation of chromosomal replication *in vivo* and *in vitro* by a mutant initiator protein, DnaAcos, of *Escherichia coli*. J Biol Chem 269:12698-12703.
5. Usongo V, Nolent F, Sanscartier P, Tanguay C, Broccoli S et al. (2008) Depletion of RNase HI activity in *Escherichia coli* lacking DNA topoisomerase I leads to defects in DNA supercoiling and segregation. Mol Microbiol 69:968-981.
6. Drolet M, Phoenix P, Menzel R, Massé E, Liu LF et al. (1995) Overexpression of RNase H partially complements the growth defect of an *Escherichia* *coli* delta *topA* mutant: R-loop formation is a major problem in the absence of DNA topoisomerase I. Proc Natl Acad Sci U S A 92:3526-3530.
7. Datsenko KA, Wanner BL (2000) One-step inactivation of chromosomal genes in *Escherichia coli* K-12 using PCR products. Proc Natl Acad Sci U S A 97: 6640-6645.
8. Broccoli S, Phoenix P, Drolet M (2000) Isolation of the *topB* gene encoding DNA topoisomerase III as a multicopy suppressor of *topA* null mutations in *Escherichia coli.* Mol Microbiol 35:58-68.
